# The Electric and Magnetic Fields Instrument Suite and Integrated Science (EMFISIS): Science, Data, and Usage Best Practices

**DOI:** 10.1007/s11214-023-00973-z

**Published:** 2023-04-25

**Authors:** C. A. Kletzing, J. Bortnik, G. Hospodarsky, W. S. Kurth, O. Santolik, C. W. Smitth, I. W. Christopher, D. P. Hartley, I. Kolmasova, A. Sen Gupta

**Affiliations:** 1 Department of Physics & Astronomy, The University of Iowa, Iowa City, USA; 2 Department of Atmospheric & Oceanic Sciences, University of California, Los Angeles, USA; 3 Institute of Atmospheric Physics of the Academy of Sciences of the Czech Republic, Prague, Czech Republic; 4 Institute for the Study of Earth, Oceans, and Space, University of New Hampshire, Durham, USA; 5 Department of Electrical and Computer Engineering, University of Iowa, Iowa City, USA

**Keywords:** Inner magnetosphere, Wave measurements, Data usage

## Abstract

We provide a post-mission assessment of the science and data from the Electric and Magnetic Field Instrument Suite and Integrated Science (EMFISIS) investigation on the NASA Van Allen Probes mission. An overview of important scientific results is presented, covering all of the key wave modes and DC magnetic fields measured by EMFISIS. Discussion of the data products, which are publicly available, follows to provide users with guidance on characteristics and known issues of the measurements. We present guidance on the correct use of derived products, in particular, the wave-normal analysis (WNA) which yields fundamental wave properties such as polarization, ellipticity, and Poynting flux. We also give information about the plasma density derived from measuring the upper hybrid line in the inner magnetosphere.

## Introduction

The Electric and Magnetic Field Instrument Suite and Integrated Science (EMFISIS) on the NASA Van Allen Probes mission provided the primary plasma waves and DC magnetic field measurements. As such it was comprised of 3 main elements: the DC fluxgate magnetometer (MAG), the Waves instrument comprising the triaxial search coil (MSC), the Wave Frequency Receiver (WFR) measuring the full vector electric and magnetic wave fields from 10 Hz to 12 kHz, and the High Frequency Receiver (HFR) measuring a single component of the electric field from 10 kHz to 500 kHz.

The full vector nature of these measurements has provided the most complete set of wave and DC magnetic field measurements ever made in the Earth’s inner magnetosphere. Of particularly note is the full 3D nature of the WFR measurements of both electric and magnetic fields. The 3D nature of these measurements allows calculation of key wave properties which is not possible without both the $\vec{E}$ and $\vec{B}$ vectors. This has enabled a wide range of scientific advances discussed below. It should be noted that the instruments on the Van Allen Probes use the UVW coordinate system which is nominally aligned all the field sensors with the W coordinate pointing along the spin axis. This was adopted to assure proper orientation of the sensors and to keep instrument coordinates distinct from spacecraft coordinates.

The EMFISIS data files are organized by 5 levels designated L0–L4. L0 are raw telemetry files. L1 are an intermediate step which only adds time tags, but the measured quantities are mostly left in telemetry units. Both L0 and L1 are not useful for scientific analysis and are not typically available to those outside the EMFISIS data processing team. L2 products are data in physical units with time stamps that have been used widely for scientific analysis. Files of this type include survey data, burst data, (spinning) magnetic field data, and instrument housekeeping data. L3 files are solely magnetic field data that have been transformed into inertial coordinates such as SM, GSM, etc. at three different time resolutions. Finally, L4 data files consist of two types – density data derived from the HFR observations of the upper hybrid (or plasma frequency cutoff, in some cases) and files with wave normal analysis (WNA) quantities. These L2–L4 data products are discussed extensively below.

In what follows, we present a review of key results using the EMFISIS measurements (Sect. 2), followed by descriptions of the MAG data (Sect. 3), WFR data including calibration and details of wave normal calculation (Sect. 4), and finally the HFR data and the plasma density derived from it (Sect. 5). We also present appendices with the mathematics of vector math in the spectral domain as well as a listing of all key instrument parameters for the EMFISIS investigation.

## Key Science Results

The primary scientific questions that the EMFISIS instrument was designed to address (Kletzing et al. 2013) included:

1. Which physical processes produce radiation belt enhancement events?
2. What are the dominant mechanisms for relativistic electron loss?
3. How do ring-current and other geomagnetic processes affect radiation belt behaviour?

In order to address these questions, a variety of waves needed to be carefully studied to accurately characterize and quantify the wave properties, morphology, excitation mechanisms, propagation and evolution, and their net effects on the energetic particle populations, both as individual waves and in concert with other wave types. The result was an explosion of magnetospheric plasma wave research, with over 500 refereed journal publications having been written in the past few years alone, directly arising from EMFISIS data, and growing daily.

The present section is an attempt to summarize some of the research and ground-breaking steps that have been made over the past few years, with the understanding that not all research studies could be included due to the sheer volume of the work and the rate at which it is being produced. Instead, a few key results have been highlighted in each section, and many others have been listed for interested readers to investigate further.

**Fig. 1:**
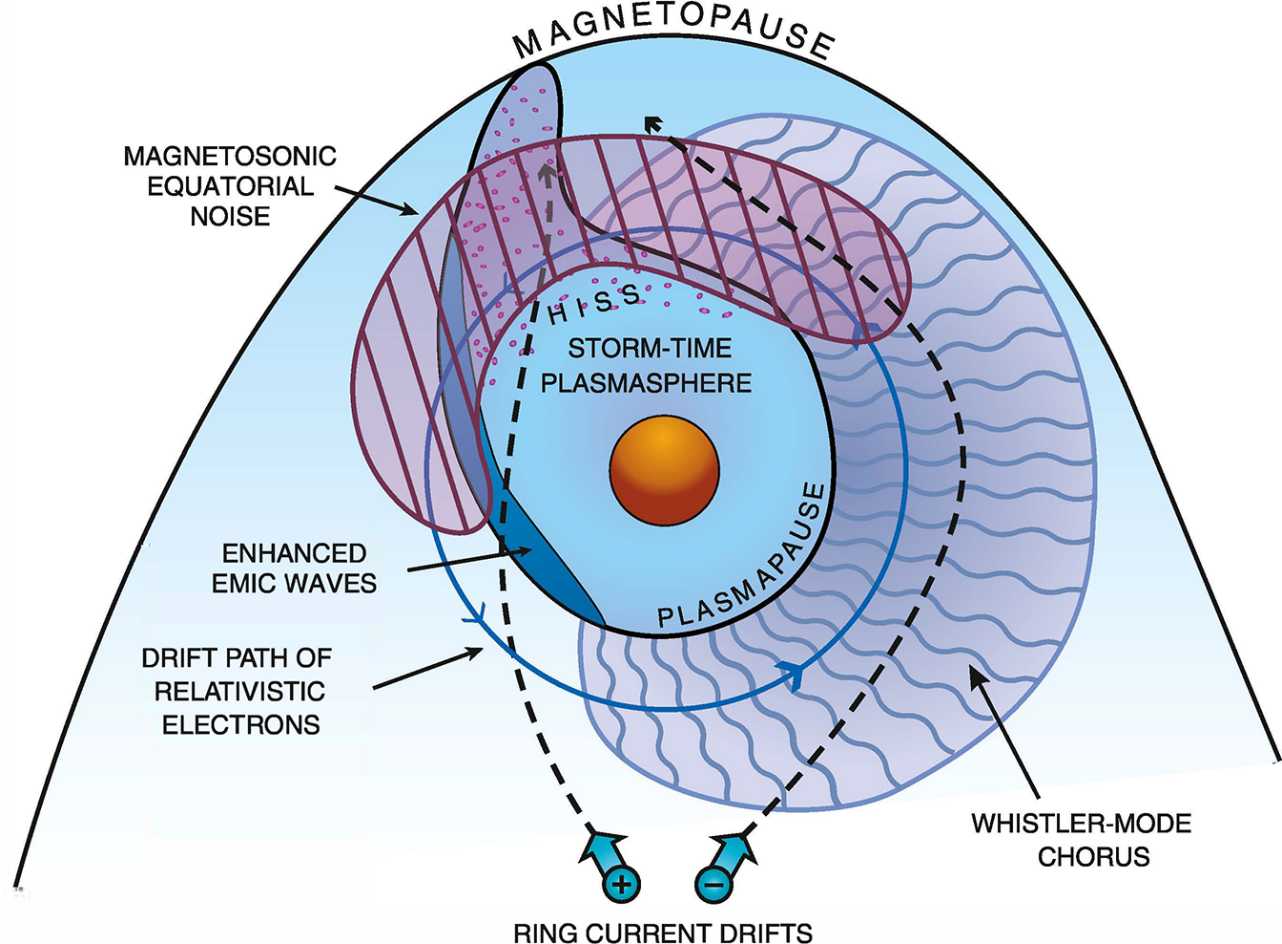
[After Thorne et al. 2010, Fig. 1]: A schematic illustration of the spatial distribution of the dominant waves in the inner magnetosphere that control the dynamics of radiation belt electrons, together with the nominal magnetopause and plasmapause locations. Superimposed are the drift trajectories of relativistic ($>0.5$ MeV) electrons and plasmasheet (10 keV) electrons and ions entering from the magnetotail

The prevailing view of inner magnetospheric physics in the years immediately leading up to the launch of the Van Allen Probes on August 30, 2012 is summarized in Fig. 1. Since the high energy particle population is essentially collisionless, particle dynamics are necessarily controlled by a combination of resonant, non-resonant, linear, and nonlinear interactions between the energetic electrons and protons, and a variety of plasma wave types. A few of these most important wave types are shown in Fig. 1 together with the spatial regions that they were believed to occupy shown in the equatorial plane. The particles drift through these wave fields, each of which exerts a different effect on the particle population, in different regions of space as well as on the particle’s energy and pitch angle, such that cumulatively the waves shape and evolve the particle populations to produce the distributions that we observe with the particle instruments on Van Allen Probes.

The complexity arises in that the spectral and spatial distributions of various wave populations can change dramatically in the course of a typical geomagnetic event, but, indeed, can also change the way they interact with the particle populations, lending further nuance to an already complicated physical process. The subsections that follow are organized in order of ascending wave frequency, beginning with the large spatial scale Ultra Low Frequency (ULF) waves in the few milliHertz (mHz) range, and ending with the 10s kiloHertz (kHz) human made Very Low Frequency (VLF) transmitter signals that represent the top of the VLF range. In all sections, a very brief introduction is provided of the wave type itself, with the focus then shifting to new research that has been made using EMFISIS data. In many cases, the boundaries of the research studies between the different waves modes, and indeed between predominantly wave and particle studies are blurry and we have taken liberties in grouping these works under particular headings which may not be fully representative of the full contents of the work. Either way, it is hoped that this review serves as a starting point and a reference to many of the wonderful scientific accomplishments that have been made with the EMFISIS data in the area of magnetospheric plasma waves, and that the reader will dive deeper into the referenced material and uncover many more studies that could not have been included in the present review.

### Ultra-Low Frequency (ULF) Waves

ULF waves are typically observed over a broad band of wave periods in the range 0.2–600 sec, placing them in the milliHertz (mHz) range of frequencies (e.g., Jacobs et al. 1964) with the higher frequency end (few Hz) of this range of waves transitioning into Electromagnetic Ion Cyclotron (EMIC) waves, discussed separately below. ULF waves can be excited by a number of sources that are either internal, or external to the magnetosphere (Menk 2011). External drivers include a solar wind shear velocity at the magnetopause boundary (Claudepierre et al. 2008) and solar wind dynamic pressure fluctuations (Ukhorskiy et al. 2006; Takahashi and Ukhorskiy 2007; Claudepierre et al. 2009; Dai et al. 2015) which can drive congressional fast-mode waves into the inner magnetosphere and are characterized by a range of wavelengths from global scale azimuthal wavelengths to relatively small azimuthal wave numbers. In contrast, internal instabilities excite more localized ULF waves with even smaller azimuthal wavelength. The instabilities could be either a drift-bounce instability (Southwood 1976; Dai et al. 2013) or drift mirror instability (Chen and Hasegawa 1991). These two instabilities, which are generally coupled, tend to be preferentially more effective in low $\beta $ and high $\beta $ plasma, respectively.

ULF waves generally redistribute the energetic particle population radially (in L shell) through a relatively slow (hours to days) radial diffusion process, or direct shock-injection of energetic electrons into the inner magnetosphere (Blake et al. 1992). It was originally believed that inward radial diffusion from high L-shells was the major source of the radiation belts (Schulz and Lanzerotti 1974) This is still believed to be the case though only at lower L-shells (Ma et al. 2015). More recent analyses have shown that during radiation belt enhancement events a localized peak develops at $\text{L}\sim 5$, indicative of a local acceleration process (Green and Kivelson 2004; Chen et al. 2006, 2007). In contrast to inward radial diffusion, it now appears that outward radial diffusion plays a major role in the initial loss of outer radiation belt electrons (during the main phase of storms) by transporting energetic electrons to the magnetopause boundary where they are permanently lost from the stable trapping region of closed magnetic field lines (Shprits et al. 2006; Bortnik et al. 2006).

Recent studies stemming from the EMFISIS data aboard the Van Allen Probes have elucidated a number of issues surrounding diffusion by ULF waves: Rae et al. (2019) has shown how the lower frequency portion of solar-wind driven ULF wave power can penetrate and accumulate closer to the Earth than is indicated in statistical wave models during geomagnetic storms compared to quiet times. Da Silva et al. (2019) showed that such ULF waves, spreading from higher to lower L-shells, were key in driving the observed inward radial diffusion that explained the recovery of the outer radiation belt during the September 22, 2014 enhancement event, and Ozeke et al. (2019) showed how the 17–18 March 2015 superstorm could be explained on the basis of fast ULF diffusive transport. Ali et al. (2016) derived a general set of such radial diffusion coefficients for energetic electrons based on Van Allen Probes data, while Selesnick et al. (2016) examined an analogous inward radial diffusion process occurring for protons.

ULF waves can impact the radiation belts directly, or by modulating other key waves such as the much higher frequency (kHz) whistler-mode chorus emissions (discussed below) deep in the magnetosphere, as demonstrated by Xia et al. (2016). The authors demonstrate that ULF waves modulate the distributions of electrons and protons, the wave intensities, and the linear growth rates consistently with the lower frequency portion of the chorus waves ($f < f_{ce}$ where $f_{ce}$ is the equatorial electron cyclotron frequency of the field line on which the spacecraft is located), but not the higher frequency portion of the lower-band chorus waves or upper-band chorus waves. This is a very interesting plasma physics problem in and of itself, and necessarily requires the action of an additional mechanism to affect that portion of the frequency spectrum.

Internal sources of ULF wave excitation have been carefully studied. For example Soto-Chavez et al. (2019) showed not only that the drift mirror instability condition was satisfied and was able to excite a ULF wave, but (for the first time) that the measured growth rate agreed with the linear growth rate prediction. Chaston et al. (2014) reported the discovery of kinetic-scale Alfvenic field-line resonances that were observed using EMFISIS during particle injections. These fluctuations had scale sizes perpendicular to the magnetic field of the order of an ion gyroradius and thus could demagnetize and accelerate ions via multiple traverses of the wave potential. Wave excitation can also occur by ion drift resonance (Dai et al. 2013) and ion drift-bounce resonance (Min et al. 2017).

### Electromagnetic Ion Cyclotron (EMIC) Waves

EMIC waves are typically found near the upper-end of the ULF wave spectrum ($\sim\text{Hz}$) and are divided into distinct frequency bands that are bounded by multiple ion gyrofrequencies at the upper frequency end (e.g., Kozyra et al. 1984). They are distinct from ULF waves in that they are generated via ion-cyclotron resonance with anisotropic ring-current ions (e.g., Cornwall 1965) near the equatorial magnetosphere in the aftermath of geomagnetic storms, and have indeed been observed to propagate only away from an equatorial excitation region which is 10 degrees wide in latitude (Loto’Aniu et al. 2005). EMIC waves are known to resonantly interact with ring current ions and relativistic electrons (e.g., Cornwall et al. 1970; Thorne and Kennel 1971; Millan and Thorne 2007) and thus act as a loss mechanism for the radiation belts, but prior to the launch of the Van Allen Probes, it was not clear what role EMIC waves played in radiation belt loss compared to magnetopause shadowing aided by rapid outward radial diffusion to the magnetopause boundary (driven by ULF waves). The key variables for quantifying the effects of these waves on the energetic particle populations are the spatial and temporal distributions of the EMIC wave activity, as well as the background plasma parameters that determine resonance energies.

In response to the above research questions, much attention has been paid to quantifying the statistical characteristics of EMIC waves using EMFISIS data, including their spatial and spectral distributions (Saikin et al. 2015, 2016; Wang et al. 2015; Chaston et al. 2018a,b), spatial coherence (Blum et al. 2016) as well as their relationship to particle injections (Remya et al. 2020, 2018; Jun et al. 2019a,b) and dependence on geomagnetic driving conditions (Engebretson et al. 2018b,a; Wang et al. 2015, 2016; Cho et al. 2016, 2017). In certain cases, it was discovered that the global EMIC wave field could span very large regions, 12 hours in local time, but narrow in L extent (Engebretson et al. 2015; Saikin et al. 2016; Yu et al. 2017b; Blum et al. 2017, 2020)

The particular role that EMIC waves play in energetic particle precipitation and loss has been extensively studied and it has been overwhelmingly demonstrated that EMIC waves cause relativistic electron precipitation that is observed both in situ (reduction in trapped particle fluxes measured on Van Allen Probes) and on low altitude monitors (Li et al. 2014d; Usanova et al. 2014; Rodger et al. 2015; Zhang et al. 2016b,a; Su et al. 2017; Yuan et al. 2018; Capannolo et al. 2018, 2019a,b; Kurita et al. 2018; Bingley et al. 2019; Nakamura et al. 2019; Sigsbee et al. 2020; Qin et al. 2018; Hendry et al. 2020). EMIC waves have also been shown to interact nonlinearly and potentially trap relativistic electrons (10s to 100s of keV), similarly to whistler waves (Artemyev et al. 2015; Chen et al. 2016) as well as bounce-resonate with high pitch angle electrons (Blum et al. 2019).

A particularly interesting question concerns the lower electron energy limit of the EMIC scattering, which has been theoretically shown to be near 1–2 MeV (Chen et al. 2011; Silin et al. 2011) and generally supported observationally (Usanova et al. 2014), indicate that this minimum resonant electron energy can extend much lower than previously believed, even reaching 250 keV (Capannolo et al. 2019a; Zhang et al. 2019c). While the exact mechanism responsible for this low energy precipitation is not fully understood, there are some indications that it may involve non-resonant wave-particle interactions (Chen et al. 2016; Denton et al. 2019). Another potential explanation involves the EMIC wave frequency approaching very close to the relevant gyrofrequency, and to that end, the spectral distribution has been statistically studied and characterized (Zhang et al. 2016b).

Among the many other interesting discoveries concerning EMIC waves, made using EMFISIS data are the observation and origin of the rare O+ band EMIC waves (Yu et al. 2015, 2017b, 2018a; Usanova et al. 2016), the spatial localization and ducting of EMIC waves (Mann et al. 2014), the observation and excitation of EMIC waves at low L-shells (Gamayunov et al. 2018; Qin et al. 2019), and the apparent ability of EMIC waves to originate from equatorial noise in the plasmasphere due to mode conversion (Miyoshi et al. 2019).

### Fast Magnetosonic (MS) Waves

Fast magnetosonic (MS) waves (sometimes referred to as Equatorial Noise emissions) are found ubiquitously over a wide range of L shells, typically $3 < L < 8$, and are generally believed to be confined within a few degrees of the equatorial plane (Boardsen et al. 2016; Ma et al. 2014). They propagate in the whistler-mode with almost perpendicular wave normal angles to the background magnetic fields, and have wave frequencies ranging from the proton gyrofrequency ($\Omega _{cp}$) to the lower hybrid resonance frequency ($\omega _{LH}$), consisting of a set of discrete emissions at harmonics of the proton gyrofrequency (Russell et al. 1970; Gurnett 1976; Santolík et al. 2004b). They are observed both within and outside of the plasmapause, and are excited by a cyclotron resonant instability with a ring distribution of energetic ions (Boardsen et al. 1992; Horne et al. 2000; Chen et al. 2010; Xiao et al. 2013).

Of particular interest to the Van Allen Probes mission, is the discovery that MS waves are able to efficiently accelerate energetic particles with timescales comparable to the other leading acceleration mechanisms, on the order of 1 day (Horne et al. 2007).

Recent studies of MS waves using EMFISIS data have focused on a number of different aspects related to excitation, distribution, and propagation of these waves. A defining characteristic of MS waves is their highly oblique wave normal angles, which was tested by Boardsen et al. (2018) and Zou et al. (2019), and indeed found to be generally true. The extreme obliquity of MS waves implies that their wave power is confined to a narrow range of latitudes near the equator, and this was found to be consistent with previous work in a study by Boardsen et al. (2016), but curiously there have been a number of reports of MS waves that have propagated far off the equator, to $\sim16\text{--}17$ degrees in latitude (Zhima et al. 2015), that appear to effectively resonate with radiation belt electrons (Ni et al. 2018).

The propagation of MS waves is an interesting topic that has received considerable attention. Teng et al. (2019) have shown that MS waves can be observed even below the proton gyrofrequency inside the plasmasphere in the noon to midnight region, despite the fact that they are generated above the proton gyrofrequency, but this is related to inward propagation from a distant source. Ma et al. (2014), Xiao et al. (2015b), Liu et al. (2018a), and Yuan et al. (2019) have all demonstrated how gradients in the plasma density (such as the plasmapause) can trap and guide MS wave power, and Ma et al. (2019a) followed up with a comprehensive global survey of MS wave power over their full frequency range.

Much has been learned about the excitation process and modulation of MS wave power. Several excitation mechanisms have been studied by Min et al. (2018), and the effects of hot protons (Liu et al. 2018b), substorm injections (Su et al. 2017), and resulting favourable conditions for their excitation have been examined by Kim and Shprits (2018). Interestingly, the MS wave power tends to exhibit fine harmonic structure that can have rising tones, or be quasiperiodically modulated (Boardsen et al. 2014; Li et al. 2017a; Němec et al. 2018, 2020; Liu et al. 2018b). This modulation has been shown to be related to a number of factors that include ULF waves (Zhu et al. 2019a; Liu et al. 2019), solar wind pressure variations, and the resulting compression and expansion of the magnetosphere (Li et al. 2017b).

The effects of MS waves on energetic particles has received considerable attention in the literature. MS waves have been shown to effectively energize not only 100s keV radiation belt electrons (often in concert with other wave types) (Ma et al. 2016; Lei et al. 2017; Hua et al. 2018), but to also heat suprathermal electrons (Horne et al. 2000) and cold ions (Yuan et al. 2018; Ma et al. 2019b) and, indeed, for such waves to be quenched by ion injections (Dai et al. 2019). An interesting consequence of the resonant interaction of MS waves with energetic electrons is the formation of a butterfly distribution in the energetic electron population (peak phase space density at pitch angles between 90 and 0 degrees) (Li et al. 2016a) and even in the ultrarelativistic electron population (Xiao et al. 2015a; Li et al. 2016a), often observed together in the slot region (Yang et al. 2017). Remarkably, such butterfly distributions have been seen to be directly modulated by MS waves by high resolution observations (Maldonado et al. 2016). A new type of MS wave occurring at low harmonic numbers has been reported by Posch et al. (2015) and has been shown to affect the energetic radiation belt electrons inside the plasmasphere (Yuan et al. 2017).

### Whistler-Mode Chorus Waves

Chorus waves are intense, right-hand elliptically polarized electromagnetic waves that propagate in the whistler-mode in the Earth’s inner magnetosphere. They are typically observed as short, coherent, chirping pulses that occur in two separate frequency bands: the lower band ($0.1f_{ce} < f < 0.5f_{ce}$) and the upper band ($0.5f_{ce} < f < f_{ce}$) with a gap in wave power at $0.5f_{ce}$ (Burtis and Helliwell 1969; Tsurutani and Smith 1974). The source of free energy for the excitation of chorus is believed to be the thermal anisotropy in the energetic electron population at $\sim30\text{--}100$ keV, which develops naturally during their transport, as electrons are injected from the tail to the inner magnetosphere during substorms. These waves are excited in the vicinity of the geomagnetic equator, in the tenuous region outside of the plasmasphere, and can accelerate the electrons in the Earth’s outer radiation belt to relativistic energies, which can act as a hazard to Earth-orbiting spacecraft.

Nearly every aspect of chorus wave physics has been thoroughly studied using the Van Allen Probes EMFISIS Waves instrument, due to its ability to observe spatiotemporal scales ranging from the microscopic to the global.

Beginning with the process of chorus wave excitation, He et al. (2015) examined the excitation and propagation of typical chorus waves, and even showed that they could be excited at L-shells as low as $\text{L}=3.5$ near the plasmapause which constitutes a new discovery (He et al. 2018a). The amplitude of the waves was shown by Xia et al. (2016) to be controlled by ULF waves that are themselves related to solar wind dynamic pressure variations (Liu et al. 2017b, 2019) while Yue et al. (2017) showed the direct response of chorus waves to interplanetary shocks. In addition, it appears that plasma density plays a key role in the amplification of both chorus and exohiss waves (discussed further below) (Zhu et al. 2018). A particularly interesting study on chorus wave excitation was carried out by Kubota et al. (2018) who focused on the generation mechanism of large amplitude, upper band chorus waves. This was unusual because typically such waves are observed to be much weaker than their lower band counterpart (Tyler et al. 2019a,b), and almost never appearing as large amplitude $\sim1$ nT intensity.

The spatial structure of the chorus excitation region is not smooth and uniform as might be initially expected but instead is rather structured and patchy. The scale size of the chorus patches in the direction transverse to the background magnetic field has been studied by several authors (Aryan et al. 2016; Agapitov et al. 2017; Teng et al. 2018; Shen et al. 2019)

Within the chorus generation region, it was shown that chorus waves were unexpectedly bimodal in wave normal angle, preferentially occurring at high and low wave normal angles (Li et al. 2016c,b; Agapitov et al. 2016; Artemyev et al. 2016; Shi et al. 2018a). While the low wave normal angle chorus waves were well-known and typically believed to be a result of cyclotron resonant interactions with unstable $>10$ keV electrons, the high wave normal chorus waves were shown to be a product of two types of resonance: cyclotron resonance with keV electrons and Landau resonance with 100–500 eV electron beams, which constituted a new finding (Li et al. 2016b). These highly oblique waves were shown to be particularly effective at particle scattering (e.g., Li et al. 2014a) and nonlinear parallel trapping of electrons in the outer radiation belt (Agapitov et al. 2015b,c).

Chorus waves were previously known to be ‘chirping’ in frequency (i.e., rapidly varying in frequency), and in a set of novel studies it was shown that the chirp rate was controlled by the background magnetic field’s inhomogeneity (Teng et al. 2017), but can, in rare cases, have very long lived, but narrow-band oscillations for up to a few 10s of seconds (Gao et al. 2017). These long-lived oscillations are seen to be related to the modulation of Langmuir waves (Li et al. 2017a). Relying on nonlinear theories of chorus wave growth, it was shown that plasma properties – density and thermal velocity – could be inferred from the chorus chirp rate measured within the generation region (Juhász et al. 2019) which is a novel application of the nonlinear growth theory and provides strong support for its validity. The chorus wave frequency distribution into an upper and lower band was closely examined in a number of studies and was explained in various ways by either the action of two different instability mechanisms (Zhou et al. 2019), a thermal anisotropy appearing at two different energy populations (Fu et al. 2014), or being self-consistently quenched at a particular energy (corresponding to $0.5f_{ce}$) as a part of the chorus wave excitation process itself (Li et al. 2019a). In addition, it was shown that during moderate to large geomagnetic storms, the lower frequency limit of chorus at $0.1f_{ce}$ was often dramatically breached, and extended to far lower frequencies which has implications for the electron energies that chorus could resonate with and hence radiation belt dynamics (Cattell et al. 2015).

The frequency chirping is a manifestation of the nonlinear nature of the wave-particle interactions that both excite the chorus waves, and control acceleration and scattering of the more energetic electrons, and such an interaction has been observed in detail (for the first time) on Van Allen Probes (Fennell et al. 2014). Such nonlinear wave-particle interactions have been examined closely by various authors (Zhang et al. 2018b, 2019b; Omura et al. 2019; Teng et al. 2018; Mourenas et al. 2018; Matsui et al. 2016).

In certain instances, nonlinear trapping of electrons by the chorus wave potential led to associated nonlinear structures (An et al. 2019) including electrostatic cyclotron harmonic (ECH) waves (Gao et al. 2018).

The nonlinear interactions were intimately tied to the frequency-time structure of the chorus wave itself and in a sequence of novel studies, it was shown that this fine structure was not a smooth and continuous frequency increase but fairly stochastic progression involving broad fluctuations of the instantaneous wave normal angle (Santolik et al. 2014a; Crabtree et al. 2017; Turner et al. 2017a) that could occasionally result in very wide frequency bands (Yu et al. 2018b).

A major topic of research in chorus wave physics is its effects on the acceleration and precipitation of energetic radiation belt electrons, acting as a conduit for transferring energy from lower energy electron populations to the MeV electron pulsations (Shklyar 2017). The starting point for quantifying both acceleration and loss is an accurate, global, time-varying model of the chorus wave power and this was developed by a number of workers, including the reconstruction of ‘event specific wave power by using the precipitation of 10s keV electrons as observed on the low-Earth orbiting POES satellites (Li et al. 2013a; Chen et al. 2014b), and parameterized models driven by solar wind and or geomagnetic indices (Agapitov et al. 2015a, 2018b; Aryan et al. 2017; Zhu et al. 2019c; Wang et al. 2016, 2019; Bingham et al. 2019).

**Fig. 2:**
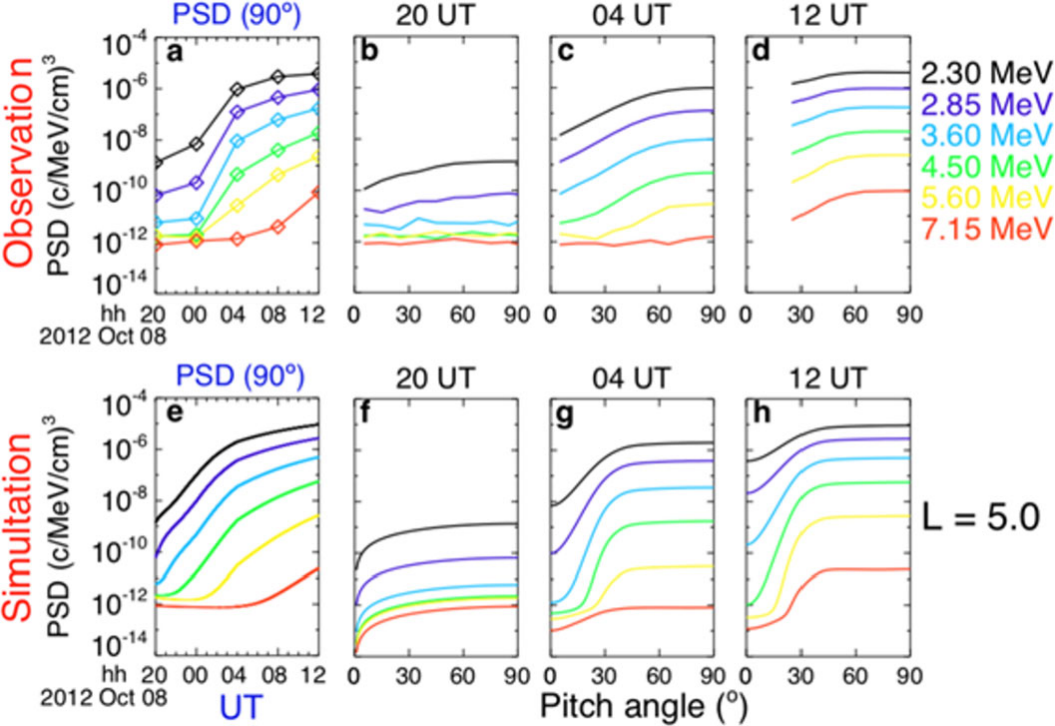
(Reproduced from Thorne et al. 2013, Fig. 4) Shows the evolution of the ultra-relativistic electron fluxes in phase space density (PSD) on October 8, 2012. (Top row) Van Allen Probes observations, and (Bottom row) diffusion based modelling with chorus as the main driver

These global wave models were subsequently used in conjunction with Fokker-Planck diffusion models to estimate the rate and characteristics of radiation belt acceleration, and it was found that chorus-driven acceleration was able to produce not only the correct timescale of acceleration, but also the correct pitch-angle distribution of energetic electrons when compared against particle measurements (Fig. 2) (Thorne et al. 2013b; Li et al. 2014a, 2016c), and (importantly) when the appropriate low cold-plasma density (measured in situ) was taken into account. The EMFISIS HFR (discussed in Sect. 5) provides regular, accurate measure of the background plasma density. Subsequent studies have shown that cold plasma density is crucial for producing fast acceleration and in some conditions could produce acceleration on timescales of 1 hour (Agapitov et al. 2019) similar to those that have been observed by others (Foster et al. 2014; Kanekal et al. 2016; Jaynes et al. 2018).

Numerous follow-up studies have confirmed the critical role played by chorus waves in accelerating electrons to relativistic (MeV) and ultra-relativistic (several MeV) energies, elucidating the controlling roles of boundary conditions, storm type (CME compared to CIR storms), injections, and other wave types such as ULF and hiss (Tu et al. 2014; Xiao et al. 2014; Li et al. 2014b; Su et al. 2014a; Liu et al. 2015; Katsavrias et al. 2015; Matsui et al. 2017; Yang et al. 2018; Hua et al. 2018; Bingham et al. 2018; Zhang et al. 2020).

Closely related to the accelerating effects of chorus waves on the seed ($\sim100\text{s}$ keV) energetic electrons (e.g., Jaynes et al. 2015b; Boyd et al. 2014; Tang et al. 2018), are the scattering effects of chorus which impact the $\sim\text{10s}$ of keV electrons which are responsible for the generation of the chorus waves themselves as well as the more energetic electrons that make up relativistic electron microbursts (e.g., Lorentzen et al. 2001; Kurita et al. 2016). The precipitation of the seed electrons has been observed by the POES satellites at Low Earth Orbit simultaneously with the coincident chorus waves observed by the EMFISIS Waves instrument on Van Allen Probes, and the ratio of the precipitating flux (0 degree channel) to the trapped flux (90 degree channel) was used as a proxy for the chorus wave intensity (Li et al. 2013a), allowing the creation of a method to recreate the so-called event-specific chorus wave intensity (Chen et al. 2014b; Tu et al. 2014; Ni et al. 2014). The energetic portion of the electron precipitation was similarly observed by Low Earth Orbiting satellites, including the recently launched FIREBIRD II (Breneman et al. 2017), and AeroCube 6B (Mozer et al. 2018).

In addition to the established topics described above, a number of chorus studies focused on novel topics such as new methods of chorus wave detection in data (Sen Gupta et al. 2017; Larchenko et al. 2019), photoelectron escape from spacecraft (Malaspina et al. 2014), relation to electron butterfly distributions (Yang et al. 2016; Jin et al. 2018), and connection to chorus observed on the ground (Demekhov et al. 2017), pulsating aurora and ULF waves (Jaynes et al. 2015a). Newer extension of chorus waves to ELF frequencies (Gao et al. 2016; Xiao et al. 2017), and the dusk side (Su et al. 2014b) have also been reported, which have challenged preconceived notions of where and how chorus waves could be observed.

### Plasmaspheric Hiss Waves

Plasmaspheric hiss waves are a population of whistler-mode waves that typically occupy the plasmasphere and plasmaspheric drainage plumes (Chan and Holzer 1976; Hayakawa and Sazhin 1992; Parrot and Lefeuvre 1986). They are found predominantly on the dayside, and respond to variations in geomagnetic activity (Thorne et al. 1973; Meredith et al. 2018). Plasmaspheric hiss waves typically tend to be incoherent and structureless, and occupy a fixed frequency band which was historically taken to be in the range $\text{f}\sim0.1\text{--}2$ kHz, but following the studies described below, this definition has been extended and our understanding of its source and characteristics significantly deepened. The proposed generation mechanisms of hiss include local instabilities and lightning (Thorne et al. 1973; Bortnik et al. 2008, and references therein) and are discussed further below. It has long been accepted that plasmaspheric hiss is responsible for both the formation of the slot region (Lyons and Thorne 1973), as well as the decay of energetic electrons in the outer radiation belt during relatively quiet times (Summers et al. 2007) due to resonant pitch angle scattering of energetic electrons (Lyons et al. 1972).

Following on from the previous section, it was theoretically shown that chorus waves could propagate from their source region outside the plasmapause, avoid intense Landau damping and leak into the plasmasphere, in the process breaking up their coherence and evolving into the hiss emission (Bortnik et al. 2008). This chorus-hiss model explained the typical characteristics of hiss, including its spatial and spectral characteristics, power and wave normal distributions, and geomagnetic dependence (Bortnik et al. 2011b,a; Chen et al. 2012a,b,c,d). A single, fortuitous observation (made prior to the launch of Van Allen Probes) between two THEMIS probes confirmed many of these theoretical predictions (Bortnik et al. 2009) but required further study to fully understand the origin of plasmaspheric hiss.

The launch of Van Allen Probes (particularly in conjunction with existing spacecraft such as THEMIS) opened the possibility for studying chorus-hiss coincident observation events on a much larger scale than was previously possible, and attain a far richer understanding of the origin of hiss. Following on from the coincident study of Bortnik et al. (2009), Li et al. (2015a) showed that chorus waves at high L-shells on the dayside, close to the magnetopause boundary, could propagate into the plasmasphere and evolve into plasmaspheric hiss, and Zhou et al. (2016) followed up with a similar coincident observation during the 3 July 2014 storm. However, the definitive study of chorus-hiss connection was performed by Agapitov et al. (2018a) who showed that there exists a region in the position dayside where chorus waves (observed outside the plasmasphere) are highly correlated to hiss waves in a statistical sense, having a delay time of a few seconds, a separation of $\sim2\text{--}4$ Earth radii and an MLT shift of $\sim1\text{--}2$ hours, all consistent with chorus acting as the embryonic source of a significant fraction of plasmaspheric hiss waves, which would then be further amplified inside the plasmasphere due to a modest local instability. The global response of hiss to solar wind driving, namely its disappearance due to interplanetary shocks and reductions in solar wind pressure appears to be quite consistent with the chorus-hiss source mechanism as shown in a number of studies (Su et al. 2015; Liu et al. 2017a,b; Chen et al. 2012b).

In contrast, Hartley et al. (2019) examined the distribution of chorus wave normal angles and found that only a very small percentage of chorus waves had the requisite wave normal angles to enter into the plasmasphere and contribute significantly to plasmasphere hiss wave power. This was true everywhere except for a small region on the dayside, which was located close to strong azimuthal gradients associated with the plasmaspheric drainage plume, where almost all of the observed chorus (94%) could theoretically evolve into hiss, leading the authors to conclude that chorus was not likely to form a substantial source of hiss wave power. Since this study contradicts the results of Agapitov et al. (2018a), a fascinating conundrum emerges about the mechanism of chorus entry into the plasmasphere.

In addition to chorus acting as the embryonic source of plasmaspheric hiss, significant evidence has begun to emerge about the localized, nonlinear growth of plasmaspheric hiss waves, supported by observation of fine frequency structure within the hiss waves themselves (Summers et al. 2014; Omura et al. 2015; Nakamura et al. 2016, 2018), and more broadly by their direction connection to electron injections from the tail (Su et al. 2018a; Zhu et al. 2019b; Liu et al. 2020).

The in situ excitation of plasmaspheric hiss by electron injections appears to trigger a new type of low-frequency (LF) hiss wave, that was observed by the Van Allen Probes at $\sim40$ Hz (Li et al. 2013b) and shown to be locally excited by a fresh injection of energetic electrons together with a propagation and recirculation effect (Chen et al. 2014a). This LF hiss emission has been statistically studied (Shi et al. 2017, 2018b) and shown to be a distinct population from the main-band plasmaspheric hiss at $\sim100\text{--}2$ kHz (Malaspina et al. 2017), and to have distinct scattering properties on the higher energy radiation belt electrons (Ni et al. 2014; Cao et al. 2017; Su et al. 2018b; Fu et al. 2020). Even more recently, it has been shown that low energy electron injections into the inner magnetosphere are able to trigger yet another new type of plasmaspheric hiss, known as high-frequency (HF) hiss, and occurring at several kHz, well above the main-band hiss wave power (He et al. 2019). The distribution, morphology, and characteristics of HF hiss have yet to be fully examined.

Due to its very well known, and long-recognized importance in scattering energetic electrons out of the radiation belts, the global distribution of hiss, including its characteristics and morphology have been studied extensively (Malaspina et al. 2016, 2018; Hartley et al. 2018b) and in a fascinating coordinated study using its precipitation, it was shown that the hiss wave power was coherently modulated over large regions of space (Breneman et al. 2015). Several global models have been developed of the spatial distribution of the hiss wave power and its various dependencies (Spasojevic et al. 2015; Yu et al. 2017a; Meredith et al. 2018), and these wave maps have been used directly to generate diffusion coefficients that feed into global simulations that quantify the radiation belt precipitation due to plasmaspheric hiss (Thorne et al. 2013a; Li et al. 2014c, 2015b; Gao et al. 2015; Ripoll et al. 2017, 2019; Watt et al. 2019; Zhu et al. 2019c; He et al. 2018b; Pinto et al. 2019; Malaspina et al. 2020).

The hiss-induced scattering of energetic electrons is typically driven by first (and higher) order cyclotron resonances, but observations showed that parallel (i.e., Landau) resonance with lower energy electrons was indeed occurring (Li et al. 2019b) and manifested itself in the form of a newly discovered reversed energy spectrum (Zhao et al. 2019).

In addition to discoveries of hiss in new frequency regions (such as LF and HF hiss), hiss also occurs in spatial regions outside of the main plasmasphere. For instance, hiss occurring in plasmaspheric drainage plumes has been shown to be surprisingly intense and effective at rapid scattering of energetic electrons (Zhang et al. 2018a, 2019a; Li et al. 2019b; Shi et al. 2019). Hiss is also frequently observed in the low-density plasmatrough region as exohiss, which is believed to be hiss that leaks out of the main plasmaspheric region (Zhu et al. 2015, 2019c; Wang et al. 2020) and can effectively scatter energetic electrons (Hua et al. 2018), although a competing source mechanism suggests that exohiss might also be formed as a result of a local three-wave interaction in this region (Gao et al. 2019)

### Lightning Generated Whistler Waves

Lightning-generated whistler (LGWs) waves were observed by ground-based VLF receivers at the very earliest stages of the space era (e.g., Helliwell 1965) and played an important role as shown in a variety of studies, including the ability to act as density probes through the course of their propagation which led to the discovery of the Earth’s plasmapause (Carpenter 1963).

In the context of radiation belt dynamics, it is not clear what role LGWs play, and how dominant that role might be. Some theoretical studies suggest that LGWs play a dominant role in controlling the lifetimes in outer region of the inner radiation belt (Abel and Thorne 1998a,b). Other studies suggest that LGWs can become unducted, magnetospherically reflect multiple times and contribute towards the plasmaspheric hiss spectrum (e.g., Draganov et al. 1992; Bortnik et al. 2002, 2003)

Recent work has shown that while lightning can indeed escape through the ionosphere, be observed in strong correlation with ground-based lightning detection systems (Zheng et al. 2016) and add to the total amount of whistler wave power in the $\sim0.5\text{--}4$ kHz range (Záhlava et al. 2019), research by Ripoll et al. (2020) shows that typically the whistler power contributed by LGWs is low compared to other sources (amplitudes of $\sim1$ pT) although in extreme events this intensity can exceed 100 pT and contribute significantly to the power below $\text{L}\sim2$. Further work is required to accurately assess where, when, and how much of an affect LGWs have on the overall radiation belt structure and dynamics.

### Very Low Frequency (VLF) Transmitter Waves

Signals from human made Very Low Frequency (VLF) transmitters that are primarily used for communications with submarines, can leak into near-Earth space and contribute to the dynamics of energetic electrons in the inner radiation belt and slot region. Narrow-band signals from ground-based VLF transmitters, the majority of which operate in the frequency range 18–27 kHz, can leak into the magnetosphere, where they then propagate in the whistler mode. The strongest wave power tends to be confined to the nightside within the region $1.2< L^{*}<2.7$ (Clilverd et al. 2008), with average peak power of the order of several $\text{pT}^{2}$ (Abel and Thorne 1998a).

The extensive spatial coverage of the inner magnetosphere allowed by the Van Allen Probes as well as the frequency coverage of the EMFISIS Waves instrument enabled the statistical study of VLF transmitter wave power and its effects on energetic electron scattering of the inner radiation belt and slot region (Ma et al. 2017; Meredith et al. 2019). It was also shown how VLF wave power is able to escape out of the ionosphere and propagate within the plasmasphere (Zhang et al. 2018c), and in a novel application it was even shown that VLF transmitter wave power could act as an effective monitor of plasmaspheric densities (Koronczay et al. 2018).

## Magnetometer (MAG) Data and Use

The EMFISIS/MAG instrument is a single-sensor fluxgate magnetometer that represents a collaboration between The University of Iowa, Goddard Space Flight Center (GSFC), and University of New Hampshire (UNH). Design and construction is detailed in Kletzing et al. (2013). Preflight calibration was performed at the GSFC magnetic calibration facility. In-flight calibration of the data was performed at UNH.

### Magnetic Cleanliness

Because the Van Allen Probes fluxgate instrument possesses only a single triaxial sensor on each spacecraft, the often-desired benefit of removing slowly varying spacecraft dipole fields offered by a dual-sensor design is not available. Calibration of the instrument means combining any offset drift with spacecraft fields and removal of the combined contribution from the measurement. This necessitates a successful magnetic cleanliness program during the spacecraft build phase to minimize and stabilize any spacecraft field at the fluxgate sensor. To accomplish this, spacecraft component designs were reviewed during the design phase where potential sources of contamination were eliminated (current loops minimized, materials reviewed, etc.). Materials, instruments, and subsystems were “sniffed” before, during and after fabrication and components with steady magnetic fields were compensated by using small permanent magnets with oppositely directed field. Both AC and DC magnetics testing of the assembled spacecraft were performed in the spacecraft assembly room (at Johns Hopkins University Applied Physics Laboratory), but not in a magnetics facility. This is made possible by careful scheduling to ensure adequate time for testing and realistic goals. The spacecraft field was required to be less than 5 nT at the sensor and this was achieved.

### Calibration

For the most accurate magnetic field measurement, it is important to know where the zero field point is for each axis (in terms of raw data values) as well as the level of orthogonality between the three axes and the overall alignment of the sensor to the spacecraft body. While pre-flight calibration provides an excellent starting point, once on orbit, variations in conditions (temperature, attitude, etc.) will cause these “zeroes” (as they are referred to by those doing magnetometer calibration) and orthogonality to drift somewhat. This leads to the need for steady, in-flight calibration of the magnetic field data.

**Fig. 3:**
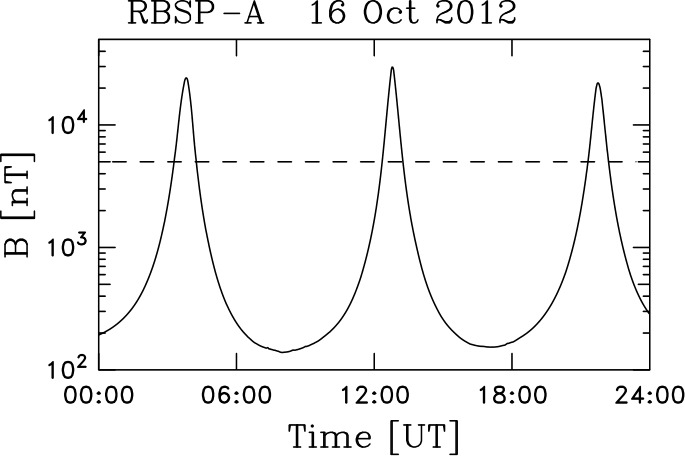
Magnetic field intensity as measured by RBSP-A on October 16, 2012. Values above $\sim 5 \times 10^{3}$ nT place the instrument into Range-3

The Van Allen Probes have a highly elliptical orbit with perigee (apogee) at 600 km (30,000 km). Approximately three orbits are completed each day. The result of the spacecraft flying through the Earth’s dipole field in this manner is that the measured magnetic field intensity changes rapidly near perigee. Figure 3 shows the measured field intensity on 16 October 2012 and is typical of the variability we see in every day of Van Allen Probes data. The MAG instrument is in Range-3 with reduced resolution on the innermost part of the orbit (when $\vert B \vert > 5 \times 10^{3}$ nT) and Range-1 throughout the rest of the orbit. The rapidly changing value of $\vert B \vert $ complicates the analysis of instrument zeroes as described by Vasquez et al. (2020). Since there is relatively less Range-3 (least sensitive) than Range-1 (higher sensitivity) data and the resolution is reduced, we perform our alignment analyses using Range-1 data only.

**Fig. 4:**
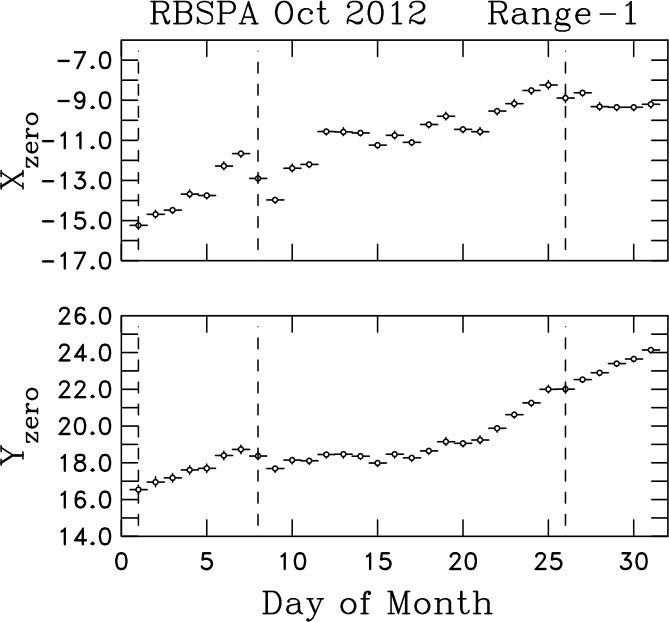
Daily averages of Range-1 instrument X- and Y-axis (spin-plane) zeroes in nT for RBSP-A during the month of October 2012. Vertical dashed lines denote days when spacecraft maneuvers were performed

The Van Allen Probes orbit precesses around the Earth with time as the Earth orbits the Sun, thereby requiring periodic pointing and orbit maneuvers to keep the solar panels properly oriented toward the Sun. We find that alignment changes are not generally significant following eclipses (which causes re-expansion of the spacecraft and booms after being in shadow), but they can be as a result of maneuvers. Figure 4 shows the calculated daily average instrument zeroes for the two spin plane axes during October 2012. There are eclipses on every day that month and maneuvers on days 1, 8, and 26 that are represented by the vertical dashed lines. The calculated zeroes for several days after the maneuvers tend to be unreliable because of spacecraft nutation. However, in this month they appear to be reasonably accurate. Each of the three maneuvers appear to mark a change in the computed zeroes. This does not explain the ramping zeroes between days 1 and 7, from days 21 and 25, and again from days 27 to 31. Also, days 27 to 31 show that the calibration of the two sensors can drift independently. Not all changes in the calibration can be attributed to maneuvers and not all maneuvers are detrimental to the desired constancy of the calibrations. The net drift in calibration zeroes over this month is $\sim 1$ nT/component. Unfortunately, background plasma fluctuations levels are frequently so low that a spin tone derived from 1 nT is significant when examining the spectra of the measured fluctuations.

Many regions of the Earth’s magnetosphere through which the Van Allen Probes pass have very low fluctuation levels compared to the mean field intensity. This magnetically quiet data places extreme demands on instrument calibration or spin tones and other noise sources stand out very clearly, particularly in spectral data. This often confuses data users who are not familiar with the spacecraft. To alleviate this confusion, significant efforts were made to calibrate MAG to the highest levels possible in order to minimize these Instrument effects. Accurate determination of the sensor alignment to better than 1 part in $10^{4}$ is equivalent to one count of MAG telemetry in Range-1, normally the most sensitive range for the instrument on this mission. This is our desired standard of calibration to minimize spin tones and is met to the best of our ability.

### Spin Tone and Interference

**Fig. 5:**
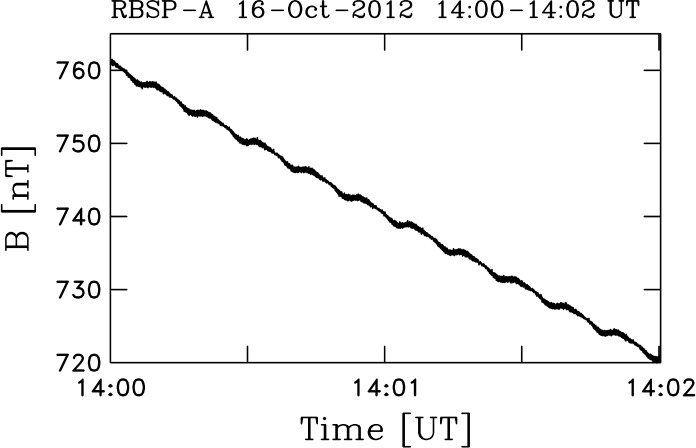
Two minutes of RBSP-A data showing $\vert B \vert $. The oscillation seen is at the spin period of the spacecraft. It is also seen in the component along the nominal spin axis of the spacecraft and in the sensor aligned with the spin axis. The spin tone exists at every stage of the processing and scales with the ambient value of $\vert B \vert $

Spin tones remain in the data (as they do in virtually all spinning spacecraft), although the are generally small. Figure 5 shows a typical example of the spin tone within the measured value of $\vert B \vert$. Examination of the spin tone amplitude during this day shows that it is a true spin-associated signal and is not generated by any spacecraft subsystem. If the spin tone arose as a result of an unknown spacecraft noise signal, an equivalent uncertainty of ten counts of raw telemetry (equivalent to $\sim 2$ nT) would be required. Figure 4 shows the day-to-day consistency of the offsets at a value that is much lower than this. While it was believed that this signal is associated with a lack of adequate knowledge of the sensor alignment with the true spin axis of the spacecraft, tests performed while writing this paper have led us to question our own analysis. Those tests are ongoing and the data will be reprocessed if an error can be found. It is also true that there are off-diagonal terms in the alignment matrix that we can only obtain to one part in $10^{3}$. This level of uncertainty is also consistent with the amplitude of the spin tone.

Cost cuts necessitated the removal of the star tracker from each spacecraft and this proved to be detrimental to MAG data quality due to a somewhat less accurate spacecraft attitude solution. This is seen in data transformed into geophysical coordinates because inaccuracy in the attitude solution introduces an effective motion of the sensor in the transformed frame. While this may not be the cause of the spin tone shown in Fig. 5, it is undeniable that the spacecraft attitude solutions suffer from this decision. Because small spacecraft motions are not completely included in the spacecraft attitude solution, precession and nutation of the spin axis as well as low frequency spacecraft oscillation (near 3 mHz) produce signals in the science data that are especially prominent in the hours to days following a spacecraft maneuver.

**Fig. 6:**
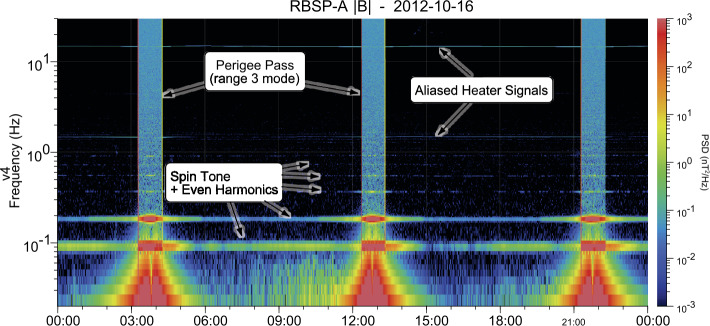
Spectrogram of $\vert B \vert $ showing spin tone and harmonics along with aliased signals that originate with the operational heater. Elevated fluctuation level when the instrument is near perigee can be attributed to the elevated noise level associated with the reduced resolution in this range

The operational heaters on the fluxgate sensors are designed to not produce a measured signal. However, with fluctuation levels as low as they are in this mission, the heater frequency is observed as an aliased signal (to lower frequency) in spectral data. Figure 6 shows the heater signal aliased at two distinct frequencies in the measured range for the same day as Fig. 5. The figure also shows the residual spin tones. Comparison of the spin tones in this figure with those in Fig. 5 show how small the heater signal is. Elevated power levels when the spacecraft is near perigee are the result of the higher instrument noise level due to reduced resolution of Range-3 measurements.

Despite the various low-level noise sources discussed above, the EMFISIS MAG data remains one of the cleanest set of magnetometer measurements in the inner magnetosphere. The EMFISIS team is presently reprocessing the MAG data and delivering version 4 of the science data. If we can improve the data further, we will reprocess the data again for delivery to the community.

## The Waves Instrument: Waveform Receiver (WFR) Data and Use

The EMFISIS Waves WaveForm Receiver (WFR) was designed to make full vector measurements of waves below the electron cyclotron frequency along the Van Allen Probes orbit through the inner magnetosphere focusing primarily on whistler mode waves outside of $\text{L}\approx 2$. In particular, the WFR was optimized for measurements of whistler-mode chorus and magnetosonic waves. A key goal of these measurements was to be able to determine wave properties such as wave normal direction, ellipticity, polarization, and Poynting flux magnitude and direction.

To achieve this goal, the WFR had a frequency response range from 10 Hz to 12 kHz for full 3D vector measurements of both the magnetic field and the electric field. The achieved response extends down to 2 Hz with an increased noise floor and is cut-off sharply at 12 kHz by the anti-aliasing filter. The data are sampled simultaneously on all six components at 35000 samples/s. This yields a Nyquist frequency at 17500 Hz and the anti-aliasing filter that cuts off sharply at 12 kHz ensures reasonable phase resolution at this cut-off frequency and excellent rejection of higher frequency signals.

The sensor for the magnetic components of the HFR is a magnetic search coil (MSC) mounted on a 3 m boom to reduce noise from spacecraft components. A very effective magnetic cleanliness program was developed which kept interference signals at extremely low level, resulting in very clean AC magnetic measurements. The electric field signals are routed to EMFISIS from the EFW experiment after differencing opposing boom pairs. They are then digitized at the same rate as the MSC signals.

### Burst Data

While survey data was sent down on a regular cadence to ensure full coverage of the entire inner magnetosphere, EMFISIS also employed a high-rate burst data scheme which sampled the data at full time resolution of 35,000 samples/sand sent the six component waveforms to the ground. All six components (3 $\vec{E}$, 3 $\vec{B}$) were sampled simultaneously. All burst intervals were 6 s (5.968 s precisely) in duration, but could be sequential with 32 ms gaps between these intervals.

Several sampling schemes were used over the course of the mission. The most common was to define a time interval which varied according to spacecraft location. Within this time interval a predefined number of the largest amplitude 6 s samples were stored and transmitted to the ground. Other sampling schemes include some “continuous” burst intervals with multiple 6 s samples one after another and a random sampling scheme executed approximately every 5 days in which 6 s samples were spread evenly over the orbit to provide sampling not biased toward the largest amplitude events.

In addition to these burst mode samples, the survey mode data is generated from a 0.5 s duration sample at the burst sampling rate. This 0.5 s sample is sent to the ground approximately every 15 minutes as a check to ensure that the on-board FFT processing produces correct results. This also provided samples throughout the orbit that were not biased toward large events

All of these burst data are available as part of the EMFISIS Level 2 (L2) archive.

### Calibration: Phase and Amplitude

An extensive series of calibrations and instrument performance checks were carried out on the EMFISIS Waves receivers and sensors, both before and after integration on the spacecraft (Kletzing et al. 2013). The basic calibration philosophy for the EMFISIS instrument was to first calibrate the receivers and sensors individually, then perform a calibration of the combined sensor and receiver systems (end-to-end). Final tests and calibrations were performed after integration on the spacecraft to verify that nothing had changed. These tests and calibrations were used to construct lookup tables that converted the telemetry data value to the true input signal strength and phase.

Amplitude calibrations for each of the EMFISIS Waves receivers and sensors were accomplished by providing an input signal of fixed frequency. The amplitude of the stimulus varied to cover the full amplitude range of the receiver. Amplitude calibrations were also performed with an input of white noise that was constant in amplitude over the frequency range of the receiver. Frequency and phase calibrations were accomplished by sweeping an input signal of known amplitude and phase over the frequency range of the receivers. For the six WFR receivers, additional calibrations were performed by applying the same white noise and a pseudo-random noise signal to the six receivers. Furthermore, due to the sharing of sensor elements (electric antennas and MSC) between the EMFISIS and EFW suites, a series of interface tests and calibrations were performed after integration on the spacecraft to verify the electrical performance and calibrations through the EFW antenna and preamplifiers to EMFISIS, and also through the MSC sensors to EFW.

These prelaunch calibrations and tests did not take into account all the possible effects due to the coupling of the plasma to the electric field antenna. In a series of papers, Hartley et al. (2015, 2016, 2022) investigated the variation in the measured wave electric fields (especially in the shorter axial spacecraft spin axis electric field antenna) compared to theoretical predictions due to the variation of the coupling due to the variation in the plasma density.

#### Six-Channel Waveform Receiver Calibration

The calibration of the WFR waveform data products have a number of specific details and processes that must be applied to correctly fully calibrate them, depending on the specific goal of the analysis. The Level 2 files provided by the EMFISIS team are calibrated in amplitude at 1 kHz only, and no phase calibrations have been applied in these files. These files include the continuous burst files (for example RBSP-a_WFR-waveform-continuous-burst_emfisis-L2_20130202T01_v1.2.5.cdf) and the survey waveform files (for example RBSP-a_WFR-waveform_emfisis-L2_20130202_v1.2.3.cdf). The flat amplitude response over most of the frequency range of the WFR channels (Kletzing et al. 2013) allow this single frequency calibration method to be useful in many studies of the waveform data. However, for some analysis, such as wave propagation studies, the full calibration needs to be applied (both amplitude and phase). The phase calibration is a frequency-dependent shift in the phase of the observed wave relative to the input wave, tantamount to a time delay at that frequency.

The L2 data can be adjusted over frequency by applying dimensionless complex factors over frequency immediately after Fourier transforming the L2 data. The file, called L2_fsw_tables_full_res_adjustment.txt, available on the EMFISIS web page and to be archived at NASA’s SPDF, consists of a table for the magnetic field (${\vec{B}}$) channels and a table for the electric field ($\vec{E}$) channels. Each table has 5600 complex entries, extending from 2.13623 to 11962.89 Hz, in steps of 2.13623 Hz. Above the highest frequency the WFR filters roll off; no calibration measurements exist, but one could apply the last value to any frequencies above that. The table was constructed assuming 16384 data points are to be Fourier transformed. If fewer than that are to be transformed, then the table can be decimated to accommodate a shorter data set. The procedure is; FFT the L2 data at the desired resolution and then perform a complex multiplication of the Fourier transformed dataset and the E or B adjustment table. It should be noted, if these results are compared to the onboard survey WFR spectra, the L2 data WFR waveform files have units of volts/meter and nanoTesla for E and B respectively, whereas the onboard survey spectra have units of RMS volts/meter and RMS nanoTesla.

### Independent Verification of WFR Timing

Lightning discharges in the Earth’s atmosphere generate powerful and impulsive radio signatures of whistlers whose frequency spectrum usually has a broad maximum in the EMFISIS Waves frequency range. These signals can be used for a verification of the absolute universal time (UT) tags of the EMFISIS Waves measurements, if we know the exact time of the source lightning stroke from an independent source.

**Fig. 7:**
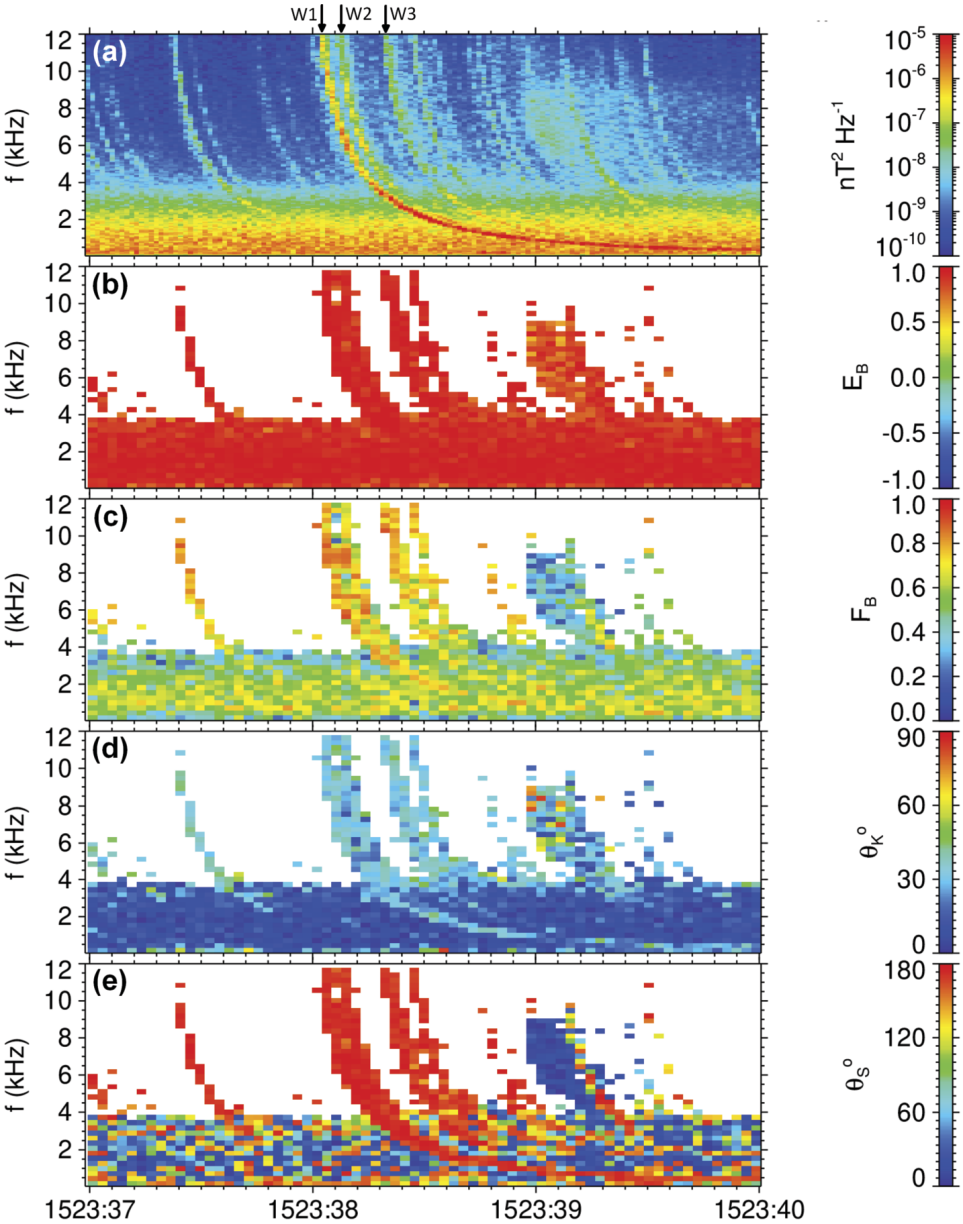
Observation of intense whistlers by the EMFISIS Waves instrument on 6 June 2013 between 15:23:37 and 15:23:40 UT: (**a**) trace of the magnetic field spectral matrix from Eq. (9), (**b**) ellipticity of the magnetic field polarization from Eq. (21) (Santolík et al. 2002), (**c**) planarity of the magnetic field polarization from Eq. (20) (Santolík et al. 2003), (**d**) inclination of the wave vector from the $\vec{B}_{0}$ direction from Eq. (17) (Santolík et al. 2003), (**e**) spectral estimate of the inclination of the Poynting vector from the $\vec{B}_{0}$ from Eq. (24) (Santolík et al. 2010). Arrows on the top show whistlers W1–W3 (see text)

Figure 7 shows a 3 s interval of a continuous waveform capture containing a sequence of strong whistlers observed by Van Allen Probe A on 6 June 2013 after 15:23:37, close to the magnetic equator at a magnetic latitude of $1.17^{\circ}$, radial distance of 2.62 $\text{R}_{E}$, and magnetic local time of 17.57 h. The frequency-time power spectrogram obtained from measurements of the EMFISIS search coil sensors (Fig. 7a) shows dispersed whistlers over the entire frequency range of the instrument up to 12 kHz, starting from a hiss band below 4 kHz. The onboard measurement by the EMFISIS fluxgate magnetometer sensor gave the electron cyclotron frequency of 44.9 kHz, well above this frequency range. The EMFISIS measurements of the upper hybrid frequency showed high plasmaspheric densities of more than 1000 $\text{cm}^{-3}$. The most pronounced whistler is marked W1 in Fig. 7a and arrives just after 15:23:38 UT. We measured the time delays to other whistlers in the sequence by determining the intervals between peaks of intensity of the whistler traces in Fig. 7a at five distinct frequencies between 6 kHz and 10 kHz. Average delays with estimates of standard deviations from these five values are $76\pm3~\text{ms}$ between the whistlers marked W1 and W2. Between whistlers W2 and W3 we find a delay of $205\pm3~\text{ms}$. Figures 7b and 7c, respectively, confirm that whistlers have a right handed circular polarization (Santolík et al. 2002) and that their magnetic field fluctuations are confined close to a single plane (Santolík et al. 2003). Their wave vector direction then can be reliably estimated (Fig. 7d), giving directions inclined by $20\text{--}35^{\circ}$ from the local magnetic field line (Santolík et al. 2003). Finally, Fig. 7e shows that the whistlers propagated to the equator from the Northern hemisphere (Santolík et al. 2010).

**Fig. 8:**
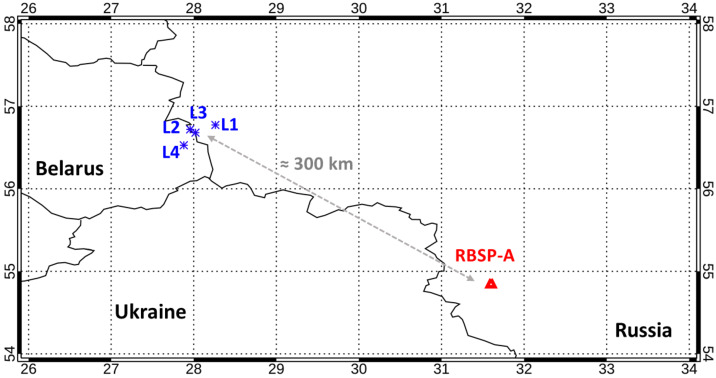
Map showing (blue) positions of source lightning discharges for whistlers L1–L4, as they were detected by the ground-based European lightning location network EUCLID; (red) magnetic footprint of Van Allen Probe A (RBSP-A)

We searched the records of the ground-based European lightning location network EUCLID for strong lightning which would occur shortly before the whistler observations, and close to the magnetic footpoint of Van Allen Probe A at geographic coordinates of $31.46^{\circ}\text{E}$ and $54.86^{\circ}\text{N}$. We found a group of four positive cloud-to-ground lightning return strokes at close locations marked L1, L2, L3, and L4 in Fig. 8, with mutual distances below 30 km and approximately 300 km from the spacecraft footpoint. This distance is also very close, well reachable for the lightning generated radio waves (atmospherics) by subionospheric propagation. The time sequence of these strokes corresponds to the sequence of whistler observations: L1 occurs at 15:23:37.5389 UT with a peak current of 63 kA, L2 strikes only 2.0 ms later with a large peak current of 119 kA, L3 with a peak current of 31 kA strikes 75.7 ms after L2, and L4 with a peak current of 51 kA strikes 205.7 ms after L3. We can then see that time delays from L2 to L3 and from L3 to L4 exactly match the above described sequence of whistlers W1, W2, and W3 recorded by Van Allen Probe A. This strongly indicates that we correctly attribute the observed whistlers to this particular set of lightning detections. The most pronounced whistler W1 in Fig. 7 then corresponds to the combined effect of the L1 stroke with the extremely strong lightning stroke L2 occurring shortly after L1.

Finally, in order to verify the timing precision between EMFISIS and EUCLID data, we assumed a ducted propagation of the whistlers along the magnetic field line to Van Allen Probe A from its magnetic footpoint. This is supported by a low inclination of the wave vectors from the local magnetic field line noted in Fig. 7d. The whistlers therefore probably propagated in a duct for most of their ray path, otherwise their wave vectors would be highly inclined. As the trajectory along the magnetic field line is approximately 2.67 $\text{R}_{E}$ long, the electromagnetic signal would travel for $t_{c}\approx 56.8~\text{ms}$ if it propagated with the speed of light. The propagation speed in a dense plasma is, however, substantially lower. In a low frequency approximation of quasi-parallel whistler propagation in dense plasmas (Helliwell 1965) we obtain a model of the arrival time,

1 $$ t = t_{0}+t_{c}+\frac{D}{\sqrt{f}}, $$

where $t_{0}$ is the time of the source lightning stroke, $D$ is the dispersion coefficient depending on the properties of the plasma medium along the wave propagation path, and $f$ is the wave frequency. We analyzed the trace of the most pronounced whistler W1 at 18 separate frequencies $f$ between 440 and 2660 Hz for the time $t$ of the maximum intensity. A least squares procedure for the model from Eq. (1) based on these 18 experimental points results in an estimate of the dispersion coefficient $D =42.6\pm 0.1~\text{s}\sqrt{\text{Hz}}$, and in an estimate of the time of the source lightning stroke $t_{0}=15{:}23{:}37.544~\text{UT}\pm 4~\text{ms}$.

Comparing now $t_{0}$ with the time, which was independently attributed by EUCLID to the strongest stroke L2 (15:23:37.540.9 UT), we come to the conclusion that both measurements match within the experimental uncertainty of $t_{0}$. As the absolute timing accuracy of EUCLID is 1 μs with respect to UT (Schulz et al. 2016), our results show that the absolute UT tags of the EMFISIS Waves measurements are correct within the 4 ms uncertainty of our analysis.

### WFR Corrections: Sheath Impedance and $\vec{E} \cdot \vec{B}$

Double probe antennas measure the potential difference between two spatially separated spherical sensors. The electric field is given as $\Delta V / L_{eff}$, where $\Delta V$ is the potential difference between the probes and $L_{eff}$ is the effective length between the sensors. For spherical double probe antennas $L_{eff}$ is typically, but not always, just the distance between the two sensors.

Whilst operating in the magnetosphere, the electric field spherical double probe sensors are electrically coupled to the plasma. This coupling can be represented by a voltage divider with complex impedance. The input voltage is given by $E L_{eff}$, but the output voltage measured by the instrument is attenuated by the voltage divider. The attenuation factor is given by impedance division, where $\Delta V_{out} / \Delta V_{in} = Z_{L} / (Z_{S} + Z_{L})$. Here $Z_{L}$ is the load impedance which is dictated by known spacecraft quantities, and $Z_{S}$ is the sheath impedance which is dictated by plasma conditions external to the spacecraft. Impedance, $Z$, is given by $1/Z = 1/R + j \omega C$, where $R$ is resistance, $j$ is the imaginary unity, $\omega $ is the angular frequency ($2 \pi f$), and $C$ is capacitance.

The electric field measured by the instrument is therefore dependent on both the instrument and plasma side of the circuit. As such, the response of electric field measurements is variable and dependent on the plasma conditions in which the antenna is operating, the frequency of the wave that is to be measured, as well as the properties of the instrumentation itself. Practically, this frequency dependent response function is not precisely known for all operating conditions leading to some degree of uncertainty in electric field wave observations.

For high frequency waves, the instrument-plasma coupling simplifies to capacitive coupling, where $\Delta V_{out} / \Delta V_{in} =C_{S} / (C_{S} + C_{L})$. If $C_{S}$ is much larger than $C_{L}$, this gives a capacitive ratio of approximately unity, meaning $V_{out} = V_{in} = E L_{eff}$. In practice however, the capacitive ratio is often closer to 0.6. For low frequency waves the resistance becomes dominant, and the signal attenuation can be approximated to $\Delta V_{out} / \Delta V_{in} = R_{L} / (R_{S} + R_{L})$. The load resistance is high, and often considered infinite. Therefore, at low frequencies, the resistance division is close to unity meaning that $V_{out} = V_{in} = E L_{eff}$. For intermediate frequencies, known as the R-C transition region between resistive and capacitive coupling, a roll off in the response of the antenna occurs. This affects both the amplitude and phase of the electric field measurements.

As previously mentioned, the effective length is oftentimes just the separation distance between the two spherical double probe sensors. This may hold for most plasma conditions frequently encountered on the Van Allen Probes orbit, however in the low-density case it may not be true. In lower densities and higher temperatures, the Debye length, $\lambda _{D}$, of the plasma increases as $\lambda _{D} = (\epsilon _{0} k_{B} T / n e^{2})^{1/2}$. If the Debye length becomes comparable to the length scale of the instrument, the effective length actually reduces to some fraction of the physical separation. This is known as a shorting factor, $s_{f}$. For the Van Allen Probes EFW instrument, the spin-axis antennas are substantially shorter than the spin plane antennas meaning that the spin-axis W component of the electric field is more susceptible to this shorting effect than the spin plane U and V components. Accounting for the shorting factor and sheath impedance means that the frequency dependent response of each antenna should vary as $s_{f} [Z_{L} / (Z_{S} + Z_{L})]$.

The frequency dependent response of electric field measurements taken by Van Allen Probes is dependent on the plasma environment in which it is operating, and dictated by the sheath resistance, the sheath capacitance, and the shorting factor. To quantify these effects, the full cold plasma dispersion relation can be applied to whistler-mode wave measurements of the magnetic field in order to predict the electric field as shown in Eq. (2). Note that it is crucial to impose appropriate thresholds for wave planarity (0.6), wave ellipticity (0.5) and wave polarization (0.5) to isolate whistler-mode waves from other wave modes prior to conducting this analysis.

2 $$ E^{2}= \frac{c^{2}}{n^{2}} \left ( \frac{ \left ( P - n^{2} \sin^{2} \theta _{k} \right )^{2} \left [ \left (\frac{D}{S-n^{2}} \right )^{2} +1 \right ] + \left (n^{2} \cos \theta _{k} \sin \theta _{k} \right )^{2} }{\left ( \frac{D}{S - n^{2}}\right )^{2} \left ( P - n^{2} \sin^{2} \theta _{k} \right )^{2} +P^{2} \cos^{2} \theta _{k}} \right ) B^{2} $$

Here, $n$ is the refractive index given by Eq. (3), $\theta _{k}$ is the polar angle of the wave vector with respect to the background magnetic field (from Singular Value Decomposition (SVD) (Santolík et al. 2003)), and $D$, $L$, $P$, $R$ and $S$ are the Stix parameters (Stix 1992).

3 $$ n^{2} = \frac{R L \sin^{2} \theta _{k} + P S \left ( 1 + \cos^{2} \theta _{k} \right ) - \sqrt{\left ( R L - P S \right ) ^{2} \sin^{4} \theta _{k} + 4 P^{2} D^{2} \cos^{2} \theta _{k}}}{2 \left ( S \sin^{2} \theta _{k} + P \cos^{2} \theta _{k} \right )} $$

This predicted electric field can then be compared to the observed value to determine the accuracy of the measured value, and to quantity the sheath properties and shorting factor. These sheath quantities can subsequently be used to correct electric field observations for instrument-plasma coupling effects.

The antenna sheath impedance for Van Allen Probes instrumentation was first investigated by Hartley et al. (2015), and quantified using this technique by Hartley et al. (2016), with the effective length shorting factor also quantified by Hartley et al. (2017). It should be noted that in these studies the sheath impedance was quantified for the sum of all three components of the electric field and not separately for each individual antenna. Additionally, the wave phase was not investigated. Hartley et al. (2022) developed a technique of using periods of favorable antenna, wave, and magnetic field geometry to quantify the antenna sheath impedance for each antenna type separately. Using time periods where the wave vector, $\vec{k}$, is approximately aligned with the background magnetic field, and the background magnetic field itself is approximately aligned with either the U or V electric field antenna directions, permits the assumption of parallel propagation and therefore the use of the simplified Eq. (4).

4 $$ E^{2}_{x, y} = \frac{c^{2}}{ \left ( 1 - \frac{f_{pe}^{2}}{f \left ( f - f_{ce}\right )}\right )} B^{2}_{y, x} $$

where $f_{ce}$ is the electron cyclotron frequency and $f_{pe}$ is inferred from the upper hybrid line (Kurth et al. 2015). Subscript $x$ and $y$ on $B^{2}$ and $E^{2}$ refer to the W and U antenna directions respectively when the V antenna is approximately aligned with the background magnetic field, and the V and W antenna directions respectively when the U antenna is approximately aligned with the background magnetic field. It should be noted that this simplified equation also assumes that $f \ll f_{pe}$ and $f_{ce} \ll f_{pe}$. For whistler-mode wave frequencies and plasma conditions this assumption is valid and yields the same results as using the full refractive index equation. From Eq. (4), the electric field can be calculated along each antenna direction during the time periods of favorable geometry, and subsequently compared to the measured electric field. This equation can be applied to the complex amplitudes of E and B in order to compare both amplitude and phase. The ratio of observed to calculated electric field is considered in Hartley et al. (2022) as a function of both frequency and plasma density for each antenna type. This analysis reveals different behaviors between the two different antenna types, which can affect the direction of the wave electric field vector, the electric field wave amplitude, as well as any parameters derived from these quantities, such as the Poynting vector.

**Fig. 9:**
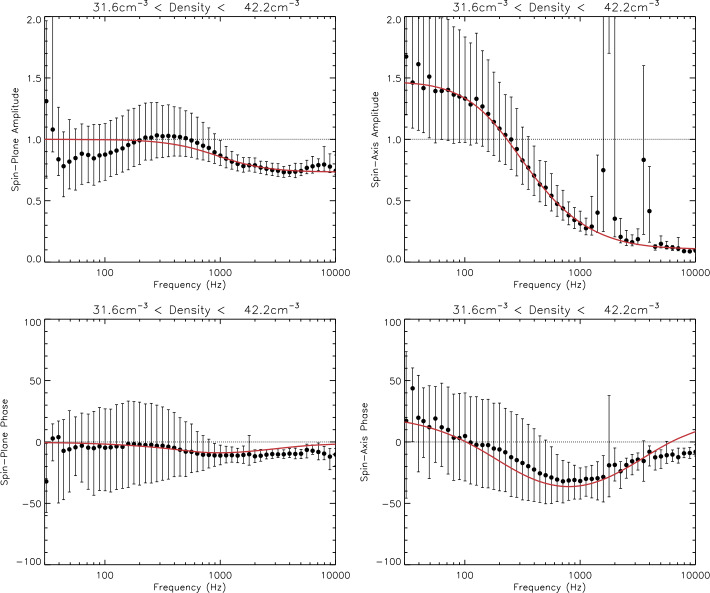
The sheath impedance corrections for (top) amplitude and (bottom) phase of the (left) spin-plane and (right) spin-axis antennas for a density between 31.6 and 42.2 $\text{cm}^{-3}$

Figure 9 shows the frequency dependent response determined through this technique of wave amplitude (top) and phase (bottom) for the spin-plane (left) and spin-axis (right) antennas for plasma density values between 31.6 and 42.2 $\text{cm}^{-3}$. The black circle symbols show the median values with the error bars indicating the 25th and 75th percentiles. Fitting to the amplitude ratios, we use the form, $s_{f} [Z_{L} / (Z_{S} + Z_{L})]$, allowing for the shorting factor and sheath properties to vary, and obtain the values that minimize the chi squared statistic. This same fitting is performed to the phase, with an additional fitting parameter to account for positive phase shifts, the cause of which are not fully understood.

This method is subsequently applied to a range of different densities to obtain the shorting factor and sheath properties as a function of plasma density. For each density, a set of fitting values are obtained based both on the amplitude and phase fits. In many cases, the amplitude and phase fits yield similar sheath properties. However, when these fits yield differing values, a decision is made as to which values shall be implemented. These sheath parameters are either manually smoothed as a function of the plasma density in order to yield a set of values to interpolate between, or, if appropriate, a simple functional form is generated. This allows for the frequency dependent response at all densities encountered on the Van Allen Probes orbit to be obtained.

For a specific density regime, the relative effective length of the spin axis antenna may be larger than unity, indicating an anomalous gain factor which is not well understood. Additionally, we are only required to account for positive phase shifts in the spin axis antenna in this same density region. This potentially indicates some missing physics that cannot be accounted for with this impedance division setup. Nevertheless, empirical adjustments are made to the sheath model in order to account for these effects.

A more complete description of this methodology, testing, the correction coefficients, as well as other notable features are provided and discussed in Hartley et al. (2022). The final sheath correction values describing the instrument response between densities of 31.6 and 42.2 $\text{cm}^{-3}$ are shown by the solid red line in Fig. 9. The black circles indicate the median ratio values, error bars indicate 25th and 75th percentiles, and the final sheath correction that is applied to the L4 data is shown by the solid red line.

**Fig. 10:**
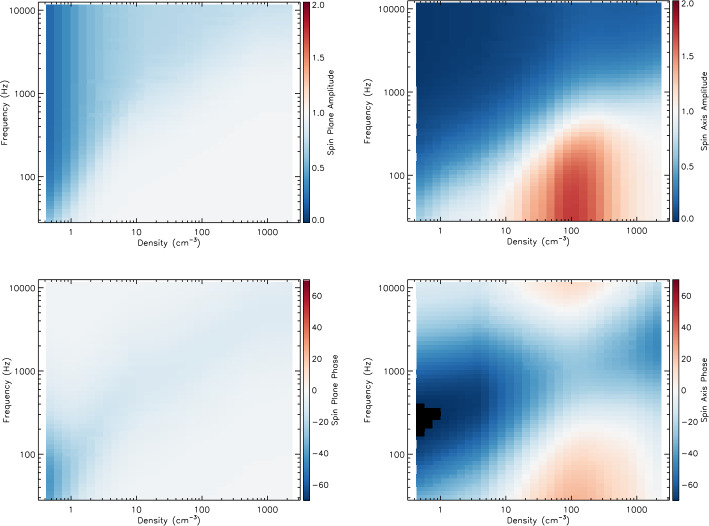
The amplitude (top) and phase (bottom) corrections to be applied to the spin plane (left) and spin axis (right) antennas

Figure 10 summarizes how the sheath impedance correction factors vary with plasma density and wave frequency for amplitude (top) and phase (bottom) for the spin-plane (left) and spin-axis (right) antennas. These sheath correction factors are subsequently applied to all electric field observations above the instrument noise floor to produce a sheath-corrected L4 dataset. Further details describing how this correction is applied and tested are provided in Hartley et al. (2022).

#### Synthesis of the Axial Electric Field

For electromagnetic waves, basic wave theory tells us that the electric and magnetic field components of the wave are orthogonal to each other and orthogonal to the direction of propagation $\hat{k}$. Given five of the six components of the wave electric and magnetic fields, the sixth component can be synthesized by using the orthogonality property: $\mathbf{E} \cdot \mathbf{B} = \mathbf{0}$.

In practice, it is rare to have the electric and magnetic field waveforms composed solely of a single electromagnetic wave mode. Consequently, one must limit the use of $\mathbf{E} \cdot \mathbf{B} = \mathbf{0}$ to frequency ranges in which the wave mode is expected to be electromagnetic, for example, whistler mode chorus propagating with a wave vector which close to aligned with background magnetic field. This criteria arises to ensure that there is a minimal electrostatic component to the wave mode. For whistler mode waves, this rules out the use of data in which the waves are propagating near the resonance cone as these can have a significant electrostatic component. Consequently, it becomes necessary to invoke the $\mathbf{E} \cdot \mathbf{B} = \mathbf{0}$ in the Fourier domain for individual frequency components. The mathematics describing this process are given in Appendix A.

This technique was applied to derive the axial $\mathbf{E}_{\mathbf{w}}$ and used as a check for the sheath corrections described above. This synthesized component is also provided as part of the sheath-corrected WNA data files.

### The Level 4 (L4) WNA Data Set: Use and Rules of Thumb

Multi-component measurements of electromagnetic waves help us to investigate their polarization properties and wave modes, their propagation in space plasmas from the source regions, and their possible interactions with particles, including nonlinear effects (Stix 1992; Gurnett and Bhattacharjee 2017). Traditional analysis methods applicable to the measurements by three orthogonal magnetic antennas were first developed for ground-based geophysical measurements (McPherron et al. 1972; Means 1972; Samson 1973; Arthur et al. 1976; Samson and Olson 1980). Investigations taking advantage of measurements of several components of the electromagnetic field on a spacecraft have been first proposed by Grard (1968) and Shawhan (1970). Traditional geophysical methods to retrieve parameters of a plane wave as well as more general wave distribution function techniques (Storey and Lefeuvre 1979, 1980; Storey et al. 1991; Kasahara et al. 1995; Santolík and Parrot 1996, 2000) have been used for analysis of measurements of spacecraft missions carrying instruments to detect alternating magnetic and electric fields by multiple magnetic search-coil antennas and with double-probe or wire electric antennas (Gurnett 1998), such as GEOS, Aureol 3, Freja, Akebono, Polar, Interball 2, Cluster, Double Star, Demeter, Themis, MMS, and Cassini. Although, these missions were designed to investigate different regions of the geospace and solar system, similar analysis methods have been used (Parrot and Lefeuvre 1986; Lefeuvre et al. 1986, 1987; Santolík and Parrot 1998, 1999; Kasahara et al. 1994; Parrot et al. 2001; Santolík et al. 2001a,b,c; Santolík and Gurnett 2002; Parrot et al. 2003; Santolík et al. 2004a; Parrot et al. 2004; Santolik 2008; Santolík et al. 2014b; Santolik et al. 2005, 2006; Taubenschuss and Santolik 2019; Turner et al. 2017b; Santolík et al. 2011). Methods that are used in the routine processing of the Van Allen Probes EMFISIS WNA data rely on this heritage.

#### Onboard FFT Analysis

Van Allen Probes EMFISIS measures the full six dimensional set of orthogonal magnetic field and electric field components sampled at $f_{s}=35$ kHz. This set is formed by three search coil signals $B_{u}$, $B_{v}$, and $B_{w}$, the two spin plane electric field antenna signals $E_{u}$ and $E_{v}$, and the spin axis antenna signal $E_{w}$. The resulting six-dimensional waveforms are either directly recorded as waveform snapshots or processed onboard by a Fast Fourier Transform (FFT) procedure with a Hann’s $\cos ^{2}$ window function, based on 0.468 s long waveform intervals of $n_{s}=16384$ samples per component, repeated every 6 s. This results in a set of six complex Fourier spectra ${\mathscr{F}_{ik}}$, $i=1\dots 6$, in $n_{s}/2$ frequency bins ($k=1\dots 8192$) with a resolution of $f_{s}/n_{s} \approx $ 2.136 Hz. In each frequency bin $k$, six complex components are composed of three dimensional complex vectors of the magnetic field and the electric field spectral amplitudes 5 in the Cartesian (u, v, w) system of coordinates, $c$ is the speed of light.

These spectra are (still onboard) processed to form averaged Hermitian spectral matrices $6\times6$ $\mathsf{S}^{\prime}_{l}$ in 65 frequency intervals:

6 $$ S^{\prime}_{ijl} = \frac{n_{s}}{n_{l}\,f_{s}} \sum _{k=b_{l}}^{e_{l}} K_{ik} \,K_{jk}^{*}\ { \mathscr{F}}_{ik}\ { \mathscr{F}}_{jk}^{*} , $$

where the symbol ^∗^ denotes the complex conjugate, the indices $i=1\dots 6$ and $j=1\dots 6$ denote the six electromagnetic field components, $K_{ik}$ and $K_{jk}$ are their frequency dependent calibration tables, and the index $l=1\dots 65$ points to one of the predefined frequency intervals (see Table 1 and Table 2) over which the spectral matrices are averaged. These intervals are defined by the FFT frequency bins $k$ ranging from $b_{l}$ and $e_{l}$, where $n_{l} = e_{l} - b_{l} -1$ is the number of FFT frequency bins in the interval $l$. The main diagonal of each matrix $\mathsf{S}^{\prime}_{l}$ provides us with the auto-spectra, i.e., power spectral densities of the six electromagnetic field components. The off-diagonal terms of $\mathsf{S}^{\prime}_{l}$ give us the cross-spectra of different components, providing us with information about their mutual phase and, when $n_{l} >1$, also about their mutual coherence. The central frequencies and bandwidths of the resulting 65 intervals are given by

7 $$\begin{aligned} \begin{aligned} f_{l} &= \frac{f_{s}}{n_{s}} \sqrt{e_{l}\ b_{l}} \\ \Delta _{l}& =\frac{f_{s}\ n_{l}}{n_{s}}. \end{aligned} \end{aligned}$$

**Table 1:** Table of semi-logarithmic frequency intervals for calculation of averaged spectral matrices according to Eq. (6). In each interval $l$, $n_{l} $ FFT frequency bins from $b_{l}$ to $e_{l}$ are accumulated. Central frequencies $f_{l}$ and bandwidths $\Delta _{l}$ are given by Eq. (7)

Interval no. (*l*)	FFT bins: $b_{l}$	$e_{l}$	$n_{l}$	$f_{l}$ [Hz]	$\Delta _{l}$ [Hz]
1	1	1	1	2.1	2.1
2	2	2	1	4.3	2.1
3	3	3	1	6.4	2.1
4	4	4	1	8.5	2.1
5	5	5	1	10.7	2.1
6	6	6	1	12.8	2.1
7	7	7	1	15.0	2.1
8	8	8	1	17.1	2.1
9	9	9	1	19.2	2.1
10	10	10	1	21.4	2.1
11	11	11	1	23.5	2.1
12	12	12	1	25.6	2.1
13	13	13	1	27.8	2.1
14	14	15	2	31.0	4.3
15	16	17	2	35.2	4.3
16	18	19	2	39.5	4.3
17	20	21	2	43.8	4.3
18	22	24	3	49.1	6.4
19	25	27	3	55.5	6.4
20	28	31	4	62.9	8.5
21	32	35	4	71.5	8.5
22	36	39	4	80.0	8.5
23	40	44	5	89.6	10.7
24	45	49	5	100.3	10.7
25	50	55	6	112.0	12.8
26	56	62	7	125.9	15.0
27	63	70	8	141.9	17.1
28	71	78	8	159.0	17.1
29	79	88	10	178.1	21.4
30	89	98	10	199.5	21.4
31	99	111	13	223.9	27.8
32	112	124	13	251.7	27.8

**Table 2:** Continuation of the table of semi-logarithmic frequency intervals for calculation of averaged spectral matrices according to Eq. (6). In each interval $l$, $n_{l} $ FFT frequency bins from $b_{l}$ to $e_{l}$ are accumulated. Central frequencies $f_{l}$ and bandwidths $\Delta _{l}$ are given by Eq. (7)

Interval no. (*l*)	FFT bins: $b_{l}$	$e_{l}$	$n_{l}$	$f_{l}$ [Hz]	$\Delta _{l}$ [Hz]
33	125	139	15	281.6	32.0
34	140	156	17	315.7	36.3
35	157	175	19	354.1	40.6
36	176	197	22	397.8	47.0
37	198	221	24	446.9	51.3
38	222	248	27	501.2	57.7
39	249	278	30	562.0	64.1
40	279	312	34	630.3	72.6
41	313	351	39	708.1	83.3
42	352	393	42	794.5	89.7
43	394	441	48	890.5	102.5
44	442	495	54	999.2	115.4
45	496	556	61	1121.8	130.3
46	557	624	68	1259.4	145.3
47	625	700	76	1413.0	162.4
48	701	785	85	1584.7	181.6
49	786	881	96	1777.7	205.1
50	882	989	108	1995.2	230.7
51	990	1110	121	2239.4	258.5
52	1111	1245	135	2512.4	288.4
53	1246	1397	152	2818.4	324.7
54	1398	1568	171	3162.8	365.3
55	1569	1759	191	3548.9	408.0
56	1760	1974	215	3981.8	459.3
57	1975	2214	240	4467.0	512.7
58	2215	2485	271	5011.9	578.9
59	2486	2788	303	5624.0	647.3
60	2789	3128	340	6309.7	726.3
61	3129	3510	382	7079.5	816.0
62	3511	3938	428	7943.3	914.3
63	3939	4419	481	8912.6	1027.5
64	4420	4958	539	10000.3	1151.4
65	4959	5600	642	11257.4	1371.5

Tables 1 and 2 define the distribution of these frequency intervals. Note that the first 13 intervals do mot involve any averaging and their bandwidth is $\Delta _{1\dots 13} = f_{s}/n_{s} \approx 2.136$ Hz. Note also that Tables 1 and 2 does not make use of all available FFT frequency indices, the last (65th) frequency interval reaching an upper frequency of $e_{65}\ f_{s} /n_{s}\approx 11.96$ kHz. This corresponds to the available frequency band below the cutoff frequency of the anti-aliasing filters of the instrument.

#### Magnetic Field Aligned Coordinates

The above described onboard processing is a basis for the EMFISIS wave normal analysis (WNA) data set which results from routine processing of two types of EMFISIS Survey mode data products, archived in the CDF format in the Level 2 (L2) database, together with the auxiliary spacecraft position and attitude data:

1. Spectral matrix data in the (u, v, w) antenna coordinate system, resulting from the onboard analysis according to Eq. (6).
2. Fluxgate magnetometer data in the (u, v, w) coordinate system.

The WNA data set has a cadence of 6 s, the same as the spectral matrix data, and a nearly 100% coverage on both Van Allen Probes. Time $t$ attributed to each data point is defined as the time of the middle of each waveform analysis interval,

8 $$ t=t_{1} + \frac{n_{s}}{2\ f_{s}} \approx t_{1}+0.234s, $$

where $t_{1}$ is the time of the first sample in the given waveform interval. To generate the WNA data the fluxgate magnetometer measurements are first linearly interpolated to time $t$, resulting in a magnetic field vector $\vec{B_{0}}$ in the (u, v, w) coordinates. The vector $\vec{B_{0}}$ is then combined with the spacecraft position and attitude data at time $t$ to derive a transformation matrix $\mathsf{M}$ from the (u, v, w) antenna coordinate system to the Cartesian magnetic field aligned (MFA) system of coordinates. The MFA coordinates have their $x_{3}$ axis parallel to $\vec{B_{0}}$ and their $x_{1}$ axis is contained in the plane defined by $\vec{B_{0}}$ and the position vector of the spacecraft with respect to the center of the Earth. The Hermitian spectral matrices $6\times6$ from Eq. (6) are then divided into four matrices $3\times3$ and transformed to the MFA system,

9 $$\begin{aligned} {S}_{ij} =& \sum _{m=1}^{3} \sum _{n=1}^{3} M_{im} M_{jn} S^{\prime}_{mn},\quad i , j =1\dots 3, \end{aligned}$$

10 $$\begin{aligned} {S}_{ij} = &\sum _{m=4}^{6} \sum _{n=1}^{3} M_{i(m-3)} M_{jn} S^{ \prime}_{mn} ,\quad i =4\dots 6 , j =1\dots 3, \end{aligned}$$

11 $$\begin{aligned} {S}_{ij} = &\sum _{m=1}^{3} \sum _{n=4}^{6} M_{im} M_{j(n-3)} S^{ \prime}_{mn} ,\quad i =1\dots 3 , j =4\dots 6, \end{aligned}$$

12 $$\begin{aligned} {S}_{ij} =& \sum _{m=4}^{6} \sum _{n=4}^{6} M_{i(m-3)} M_{j(n-3)} S^{ \prime}_{mn} ,\quad i , j =4\dots 6, \end{aligned}$$

where we drop index $l = 1\dots 65$ for simplicity, knowing that the same transformation is done for spectral matrices in all the 65 frequency intervals of a given waveform analysis interval. Equations (9) and (12) separately describe the transformation of Hermitian matrices of the magnetic and electric field components. Equations (10) and (11) refer to the transformation of mixed matrices of the magnetic field and the electric field components, with matrix (11) being simply a complex conjugate of the matrix (10) in both coordinate systems. By combining the four transformed matrices from Eqs. (9)–(12) we obtain a $6\times6$ Hermitian matrix $\mathsf{S}$, which is used for further analysis.

#### Estimation of the Wave Vector Direction

Assuming the presence of a single plane wave at a frequency $f$ with a wave vector $\vec{k}$, and assuming further the absence of experimental noise on estimates of the complex magnetic field spectral amplitude  and the electric field spectral amplitude , we can rewrite the Faraday’s law 13 A consequence of this equation is that  is always perpendicular to both $\vec{k}$ and , 1415

If we now write the complex scalar equation (14) in the Cartesian MFA coordinates and multiply it successively by three Cartesian components of the complex conjugate vectors  we obtain, with the averages according to Eq. (6) and coordinate transformation according to Eq. (9), a homogeneous set of three complex equations, which can be written as six real equations,

16 $$ \mathsf{A}\cdot \vec{k} = \left ( \textstyle\begin{array}{r @{\quad}r @{\quad}r} \Re S_{11} & \Re S_{12}& \Re S_{13} \\ \Re S_{12} & \Re S_{22}& \Re S_{23} \\ \Re S_{13} & \Re S_{23}& \Re S_{33} \\ 0 & -\Im S_{12}& -\Im S_{13} \\ \Im S_{12}& 0 & -\Im S_{23} \\ \Im S_{13}& \Im S_{23} & 0 \end{array}\displaystyle \right ) \cdot \left ( \textstyle\begin{array}{r} k_{1} \\ k_{2} \\ k_{3} \end{array}\displaystyle \right ) = 0, $$

where $\Re $ means the real part, $\Im $ means the imaginary part, and where $S_{ij}$ are components of a Hermitian spectral matrix from Eq. (9) for $i, j=1\dots 3$ and for a frequency interval $l$.

In the MFA coordinates the wave vector direction can be defined by two angles $\theta _{K}$ and $\phi _{K}$, where $\theta _{K}$ is the deviation from the $\vec{B}_{0}$ direction and $\phi _{K}$ is an azimuth centered on the plane of the local magnetic meridian). The wave vector from Eq. (16) then reads

17 $$ {\vec{k}} = k\,(\sin \theta _{K}\cos \phi _{K},\sin \theta _{K}\sin \phi _{K},\cos \theta _{K} )\, . $$

Equation (14) does not allow us to determine the modulus $k$, consistent with obtaining a homogeneous system given in (16). We therefore only have two real unknowns $\theta _{K}$ and $\phi _{K}$. In the idealized case, assuming the strict validity of the assumptions of Eq. (13), the set of Eqs. (16) hence reduces to only two independent real equations corresponding to the single complex Eq. (14). The expansion to six equations in (16) becomes, however, important when it is used with experimental data from Eq. (9). In this case, random experimental noise together with deviations of natural wave fields from a planar polarization means that the assumptions of Eq. (13) are not strictly valid.

As a consequence, all six equations in the system (16) should be taken into account. The EMFISIS WNA dataset makes use of the singular value decomposition (SVD) technique to estimate a solution of the entire set of Eqs. (16) using the analysis method as described in detail in Santolík et al. (2003). The matrix $\mathsf{A}$ is decomposed

18 $$ \mathsf{A} = \mathsf{U}\cdot \mathsf{W}\cdot \mathsf{V}^{\mathrm{T}}, $$

where $\mathsf{U}$ is a matrix $6\times3$ with orthonormal columns, $\mathsf{W}$ is a diagonal matrix $3\times3$ of three non-negative singular values $w_{3} \ge w_{2} \ge w_{1}$, and $\mathsf{V}^{\mathrm{T}}$ is a matrix $3\times3$ with orthonormal rows. The estimate of $\theta _{K}$ and $\phi_{K}$ is then found by replacing $\vec{k}$ in Eq. (17) by the column of $\mathsf{V}$ corresponding to the minimum singular value $w_{1}$. This solution therefore represents a least-squares estimate which takes into account all six equations from the set (16) and hence all the components of the experimentally obtained magnetic field spectral matrix (9). The EMFISIS WNA data set comprises the SVD estimates of $\theta_{K}$ and $\phi _{K}$, as well as other quantities presented in Table 3.

**Table 3:** Table of quantities from Santolík (2003) contained in the EMFISIS WNA data set, as functions of time and frequency. Time (epoch) is defined as $t$ from Eq. (8) based on regular 0.468 s long waveform intervals analyzed onboard with a 6-s cadence. The set of 65 frequency intervals used for this analysis is defined in Tables 1 and 2. Examples are given in separate panels of Figs. 11 and 12

Quantity	Description	Original reference	Fig.
bsum	$S_{B}$: trace of the magnetic field spectral matrix, $S_{11} +S_{22} +S_{33} = S^{\prime}_{11} +S^{\prime}_{22} +S^{\prime}_{33}$ from Eq. (9)	–	11(b)
ellsvd	$E_{B}$: ellipticity of the magnetic field polarization from Eq. (21)	*L* in paragraph 43 of Santolík et al. (2002)	11(c)
polsvd	$C_{B}$: two-dimensional degree of magnetic field coherence in the polarization plane	$C_{B}$ in Fig. 1(g) of Santolík and Gurnett (2002)	11(d)
thsvd	$\theta _{K}$: inclination of the wave vector from the $\vec{B}_{0}$ direction from Eq. (17)	*θ* in Eq. (9) and Eq. (10) of Santolík et al. (2003), paragraph 13 of Santolík et al. (2003)	11(e)
phsvd	$\phi _{K}$: azimuth of the wave vector measured in the plane perpendicular to $\vec{B}_{0}$ and centered on the plane of the local magnetic meridian from Eq. (17)	*ϕ* in Eq. (9) and Eq. (10) of Santolík et al. (2003), paragraph 13 of Santolík et al. (2003)	11(f)
plansvd	$F_{B}$: planarity of the magnetic field polarization from Eq. (20)	*F* in Eq. (12) of Santolík et al. (2003)	11(g)
esum	$S_{E}$: trace of the electric field spectral matrix, $S_{44} +S_{55} +S_{66} = S^{\prime}_{44} +S^{\prime}_{55} +S^{\prime}_{66}$ from Eq. (12)	–	12(b)
plansvde	$F_{E}$: electromagnetic planarity	$F_{E}$ from Eq. (24) of Santolík et al. (2003)	12(c)
poy1_2_3	$S_{S}$: spectral density of Poynting flux from Eq. (24)	$S_{S}$ from Eq. (12) of Santolík et al. (2010)	12(d)
thpoy1_2_3	$\theta _{S}$: spectral estimate of the inclination of the Poynting vector from the $\vec{B}_{0}$ from Eq. (24) direction	$\theta ^{\prime}_{S}$ from Eq. (13) of Santolík et al. (2010)	12(e)
phpoy1_2_3	$\phi _{S}$: spectral estimate of the azimuth of the Poynting vector measured in the plane perpendicular to $\vec{B}_{0}$ and centered on the plane of the local magnetic meridian from Eq. (24)	Equation (12) of Santolík et al. (2010)	12(f)

Since the method is only based on Eq. (14) it is unable to distinguish two anti-parallel wave vector directions (Santolík et al. 2003): both are perpendicular to the same plane. Each solution for $\theta _{K}$ and $\phi _{K}$ therefore naturally comes also with its anti-parallel direction,

19 $$ \theta _{K}^{\prime}=180^{\circ} - \theta _{K}, \quad \phi _{K}^{\prime}=180^{o} + \phi _{K}. $$

Using only the magnetic field spectral matrix from Eq. (9) both solutions ($\theta _{K}$, $\phi _{K}$) and ($\theta _{K}^{ \prime}$, $\phi _{K}^{\prime}$) are equally valid. Involving other parts of matrix $\mathsf{S}$ is thus necessary to distinguish between these two solutions, as it will be discussed later in Sect. 4.5.6.

#### Reliability of the Wave Vector Data: The Planarity Estimator

The EMFISIS WNA data set also includes the SVD estimate of the planarity of the magnetic field polarization (Santolík et al. 2003),

20 $$ F_{B} = 1-\sqrt{\frac{w_{1}}{w_{3}}}, $$

which allows us to quantify the closeness of the measured wave magnetic field to the planar polarization. A value $F_{B}=1$ would correspond to an idealized situation of exactly planar polarization without any noise, while $F_{B}=0$ would be obtained for an ideal spectral matrix of a completely randomly polarized noise without any preferred direction.

In practice, the value of $F_{B}$ is sensitive to the number of spectral matrices which are averaged before the SVD analysis. Without any averaging, for example in the first 13 frequency intervals of the EMFISIS WNA data set in Tables 1 and 2, the spectral matrix is obtained as absolutely coherent, with the modulus of cross-spectra equal to the geometric average of the corresponding auto-spectra, $|S_{ij}| = \sqrt{S_{ii}\,S_{jj}}$. In that case, one of the singular values is always zero. Hence, according to Eq. (20), $F_{B}=1$ even for a randomly polarized spectral matrix of isotropic noise. Averaging of spectral matrices is therefore necessary for this estimator.

As the presence of a single plane wave is assumed in the above described calculation of the wave vector direction, the validity of this approximation needs to be always tested. The SVD planarity estimator is then a natural choice for this test. However, different wave modes and different experimental situations impose different threshold values of $F_{B}$ which must be therefore carefully considered on case-by-case basis. For example, for an ideal elliptically polarized plane wave in the presence of noise (Taubenschuss and Santolik 2019), a planarity threshold $F_{B}>0.8$ leads to uncertainties of less than $5^{o}$ in the determination of $\theta _{K}$ for $n_{s}=7$ averaged spectral matrices. When we average $n_{s} \ge 100$ spectral matrices, a lower threshold of $F_{B}>0.5$ is sufficient for the same $5^{o}$ uncertainty in the determination of $\theta _{K}$. Note that the condition $n_{s}\ge 7$ is reached at frequencies above 120 Hz in Tables 1 and 2, and $n_{s}\ge 100$ is used at frequencies above 1.9 kHz.

Another idealized case for the planarity tests is the situation when the wave field is composed of several superposed plane waves with random mutual phases but no noise is present. A Z-mode simulation of ideal spectral matrices (Santolík et al. 2003) shows that a characteristic width of $\sim15^{\circ}$ for a wave distribution function according to a Gaussian model (Santolík and Parrot 2000) leads to a decrease of the planarity down to a value of $F_{B}\approx 0.8$. Note that the obtained results for $\theta _{K}$ and $\phi _{K}$ in this case do not necessarily retrieve the mean direction of the Gaussian model and that this systematic bias logically increases with the characteristic width of the distribution, depending also on the properties of the particular wave mode.

Note also that Eq. (20) is based only on the magnetic field spectral matrix from Eq. (9). Therefore, $F_{B}$ is unable to distinguish two anti-parallel directions: any combination or two plane waves with ($\theta _{K}$, $\phi _{K}$) and ($\theta _{K}^{\prime}$, $\phi _{K}^{\prime}$) from Eq. (19) would result in $F_{B}=1$. To recognize that such a combination violates the initial plane wave assumption we again need to involve other parts of matrix $\mathsf{S}$ (see Sect. 4.5.6).

#### Estimation of the Wave Mode

As the plasma medium allows propagation of more wave modes with different wave lengths and different polarization at a given frequency (Stix 1992; Gurnett and Bhattacharjee 2017), results obtained in separate frequency bins from the Tables 1 and 2 may belong to different modes.

To recognize the modes in which the waves propagate, the EMFISIS WNA data set contains an SVD estimator of the ellipticity of the magnetic field polarization (see Santolík et al. 2002) obtained from two largest singular values and from the sign of the imaginary component of the cross-spectrum of the two magnetic field components perpendicular to $\vec{B}_{0}$:

21 $$ E_{B} = \frac{\Im S_{12}}{|\Im S_{12}|}\,\frac{w_{2}}{w_{3}}. $$

The obtained values are therefore between −1 and 1 and, in our convention, the interpretation of special cases is as follows:

1. $E_{B} = +1$ for the right handed circular polarization (typical, for example, for the whistler mode waves or for the free space R-X mode waves propagating along the magnetic field lines),
2. $E_{B} = -1$ for the left handed circular polarization (typical, for example, for the ion cyclotron waves or for the free space L-O mode waves propagating along the magnetic field lines), and
3. $E_{B} = 0$ for the linear polarization (typical, for example, for the X mode whistler mode waves below the lower hybrid frequency propagating perpendicular to the local magnetic field line).

All other possible results indicate elliptical magnetic field polarization, either right-handed (for $E_{B} >0$) or left-handed (for $E_{B} <0$).

The reliability of the determination of the wave mode is influenced by the coherence of the measured magnetic field fluctuations. Random phase shifts between their components can be caused by experimental noise or by the presence of different modes and wave vector directions at the same time and frequency. A measure of this randomness in the EMFISIS WNA data set is the 2-D degree of coherence $C_{B}$ in the polarization plane of the wave magnetic field (Santolík and Gurnett 2002; Santolík et al. 2002). It is obtained by a transformation of the magnetic spectral matrix $S_{ij}$ for $i, j=1\dots 3$ for each frequency interval $l$ from Eq. (9) into the coordinates linked to the ellipsoid of polarization. This transformation can be done using the orthonormal matrix $\mathsf{V}$ from the SVD analysis in Eq. (18), after its columns are reordered by the corresponding singular values ($w_{3} \ge w_{2} \ge w_{1}$),

22 $$\begin{aligned} \begin{aligned} \mathsf{R} & = \mathsf{V^{T}} \cdot \mathsf{S} \cdot \mathsf{V}, \\ C_{B} &= \sqrt{2 \frac{R_{22}^{2}+R_{33}^{2}+2|R_{23}|^{2}}{\left (R_{22}+R_{33}\right )^{2}} -1}. \end{aligned} \end{aligned}$$

For an ideally coherent wave with $|R_{23}| = \sqrt{R_{22}\,R_{33}}$ we obtain $C_{B}=1$, while for idealized random noise matrix with $R_{23}=0$ and $R_{22}=R_{33}$ Eq. (22) gives $C_{B}=0$. In reality, we observe the same effect as for the planarity estimator from Eq. (20): the value of $C_{B}$ for random noise depends on the number of averaged spectral matrices. For example, in the first 13 frequency intervals of the EMFISIS WNA data set in Tables 1 and 2 (frequencies below 30 Hz), no averaging is done. The analyzed spectral matrix is then artificially obtained as absolutely coherent, and we obtain a trivial result $C_{B}=1$ which does not reflect real properties of the analyzed waves.

#### Estimation of the Poynting Vector and Electromagnetic Planarity

As discussed above in Sect. 4.5.3, the ambiguity of wave vector direction from Eq. (19) can be resolved by analyzing the parts of the spectral matrix $\mathsf{S}$ which include the electric field measurements (Eqs. (10)–(12)). Power carried by a propagating electromagnetic wave at a frequency $f$ can be described by the Poynting vector, which reads 23 where  represents the reactive power, while the real power is given by . After averaging the real part of the Poynting vector into the frequency intervals in Eq. (10) we obtain, after Santolík et al. (2010), its spectral density in the MFA coordinate system,

24 $$\begin{aligned} \begin{aligned} S_{S}\,\sin \theta _{S}\cos \phi _{S} &= \displaystyle{ \frac{1}{\mu _{0}}} \left ( \Re S_{53} - \Re S_{62} \right ) \\ S_{S}\,\sin \theta _{S}\sin \phi _{S}&= \displaystyle{ \frac{1}{\mu _{0}}} \left ( \Re S_{61} - \Re S_{43} \right ) \\ S_{S}\,\cos \theta _{S}&=\displaystyle{\frac{1}{\mu _{0}}} \left ( \Re S_{42} - \Re S_{51} \right ), \end{aligned} \end{aligned}$$

where $S_{S}$ is the spectral density of the Poynting flux, $\theta _{S}$ is a spectral estimate of the inclination of the Poynting vector from the $\vec{B}_{0}$ direction, and $\phi _{S}$ is a spectral estimate of the azimuth of the Poynting vector measured in the plane perpendicular to $\vec{B}_{0}$ and centered on the plane of the local magnetic meridian with $0^{\circ}$ pointing radially outward from the Earth. Obtained values of $\theta _{S}$ can be then used to resolve the two antiparallel solutions for the wave vector described by Eq. (19).

Calculation of the spectral estimate of the Poynting vector, however, does not rely on the assumption of the presence of a single plane wave. It simply provides us with a summary result for a particular distribution of electromagnetic waves with different wave vector directions or propagation modes. The spectral estimate of the Poynting vector is therefore unable to test the plane wave hypothesis in a strict sense, i.e., to recognize also the anti-parallel directions which degenerate into a single solution with the methods based only on the magnetic field spectral matrix from Eq. (9), as for example in Eq. (20) for the magnetic field planarity estimator. Unlike in that case, we also must make use of the measurement of both the magnetic field and the electric field.

A suitable technique is based on Eq. (13), which can be rewritten in a similar way as we did for Eq. (16). At each frequency we now multiply the three complex Eqs. (13) for the three components in the MFA system by a complex conjugate of the six dimensional vector  from Eq. (5). With the averages according to Eq. (6) and coordinate transformation according to Eqs. (9)–(12) we obtain a set of 18 complex equations for the components of the $6\times6$ spectral matrix $\mathsf{S}$:

25 $$ \frac{c}{2\pi f}\,\sum _{j,k=1}^{3} \epsilon _{ijk}\, S_{k+3,l}\, k_{j} = S_{il},\quad i=1\dots 3,\ l=1\dots 6, $$

where $\epsilon _{ijk}$ is the Levi-Civita permutation symbol. This corresponds to a system of 36 real equations which must all be satisfied if the assumptions of Eq. (13) are valid, i.e. if matrix $\mathsf{S}$ corresponds to the electromagnetic field of a single plane wave without any noise:

26 $$ \frac{c}{2\pi f}\, \mathsf{A}_{E}\cdot \vec{k} = \vec{b}, $$

where the $\mathsf{A}_{E}$ is a real matrix $36\times3$ and $\vec{b}$ is a real vector of 36 components, both derived from Eq. (25) in a straightforward way.

The electromagnetic planarity is then defined according to Santolík et al. (2003),

27 $$ F_{E} = 1 - \sqrt{ \frac{\sum _{i=1}^{36} \left (\frac{c}{2\pi f}\sum _{j=1}^{3} A_{Eij}\,k_{j} - b_{i} \right )^{2}}{\sum _{i=1}^{36} \left (\frac{c}{2\pi f}\left |\sum _{j=1}^{3} A_{Eij}\,k_{j}\right | + \left | b_{i}\right | \right )^{2}}}, $$

where $\vec{k}$ is obtained from the SVD solution of the overdetermined system (26). A value close to $F_{E} = 1$ then corresponds to the presence of a single plane wave. Lower values indicate waves coming from different directions at the same time, including anti-parallel propagating waves, which cannot be distinguished by the magnetic planarity estimator $F_{B}$ from Eq. (20).

The advantage of this procedure is that the transformed matrix of Eq. (10) can be easily used to obtain the Poynting vector and electromagnetic planarity without the need to estimate the spin-axis component of the electric field from a plane wave approximation. A disadvantage, which should be carefully taken into account when interpreting the EMFISIS WNA data is that the measurements of the two spin plane (u, v) electric field antennas are in Eqs. (10)–(12) inevitably mixed with the measurements of the spin axis (w) electric field antenna. This antenna has worse noise properties than the spin plane antenna and all antennas degraded with time toward the end of the Van Allen Probes mission. It also has different density-dependent coupling to the surrounding plasma (see Sect. 4.4). This is not included in the onboard calibration procedure (6) and may therefore significantly influence all analysis results which involve the electric field measurements, i.e., the power spectral density of the electric field, the Poynting vector and the electromagnetic planarity.

#### Example of the EMFISIS WNA Data

**Fig. 11:**
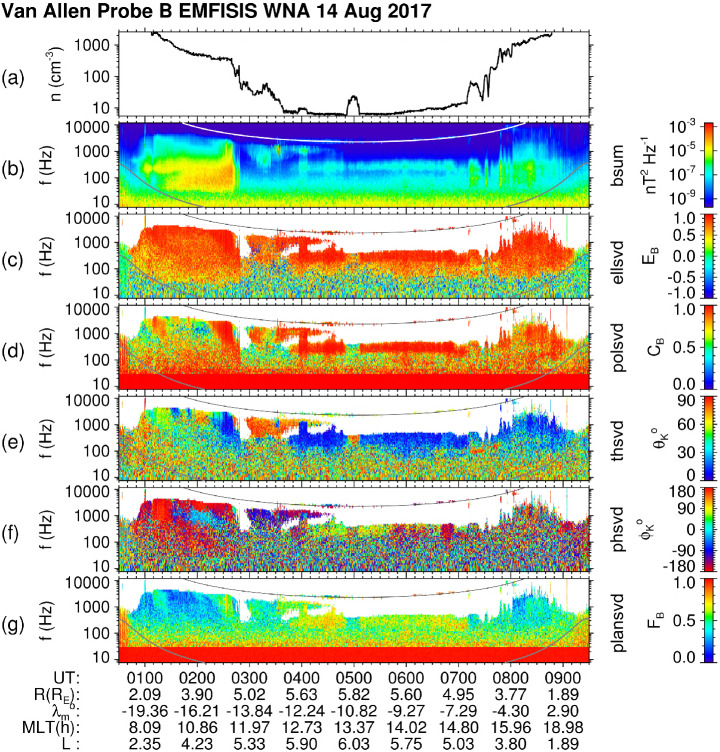
Example of the EMFISIS WNA data for one orbit of the Van Allen Probe B on 14 August 2017 between 0:30 and 9:30 UT. Panel (**a**) shows the plasma density estimated from the EMFISIS observations of the upper hybrid frequency (Kurth et al. 2015). Quantities shown in panels (**b**–**g**) are described in Table 3. White or black curves in these panels represent one half of the electron cyclotron frequency from the measurements of the EMFISIS flux-gate magnetometer. Grey curves show the lower hybrid frequency estimated from the same measurements assuming that the plasma frequency is much larger than the cyclotron frequency. Plotting threshold for panels (**c**–**g**) is $S_{B}>3\times 10^{-9}$. Spacecraft coordinates are given on the bottom: radial distance ($R$), magnetic dipole latitude ($\lambda _{m}$), magnetic local time (MLT) and the McIllwain’s parameter (L)

Figure 11 shows an example of the WNA results based on the magnetic field measurements. Geophysical context is given by the plasma density estimated using the method of Kurth et al. (2015) and by the measurements of the background magnetic field $\vec{B_{0}}$. The presented EMFISIS WNA data are summarized in Table 3 with references to separate panels of Fig. 11. The time interval goes from the perigee on the night side through the dawn side, with the dayside apogee in the middle of the analyzed interval and then back to the perigee through the dusk side sector. Right-hand polarized whistler mode emissions of plasmaspheric hiss are initially observed at lower radial distances inside the plasmasphere with low coherence and planarity implying mixed wave vector directions. Close to the dawn-side plasmapause, hiss becomes well coherent and its wave vector becomes aligned with the local magnetic field line. On the outward edge of the dawn-side density gradient exohiss and chorus emissions show high wave vector angles, followed by a weak field aligned exohiss at apogee. During the dusk-side reentry into the plasmasphere bursty lightning whistlers are observed, both ducted and unducted. Note that unrealistic unity values discussed in Sects. 4.5.5 and 4.5.4 appear at the lowest frequencies for the degree of coherence $C_{B}$ (Fig. 11d) and planarity $F_{B}$ (Fig. 11g).

**Fig. 12:**
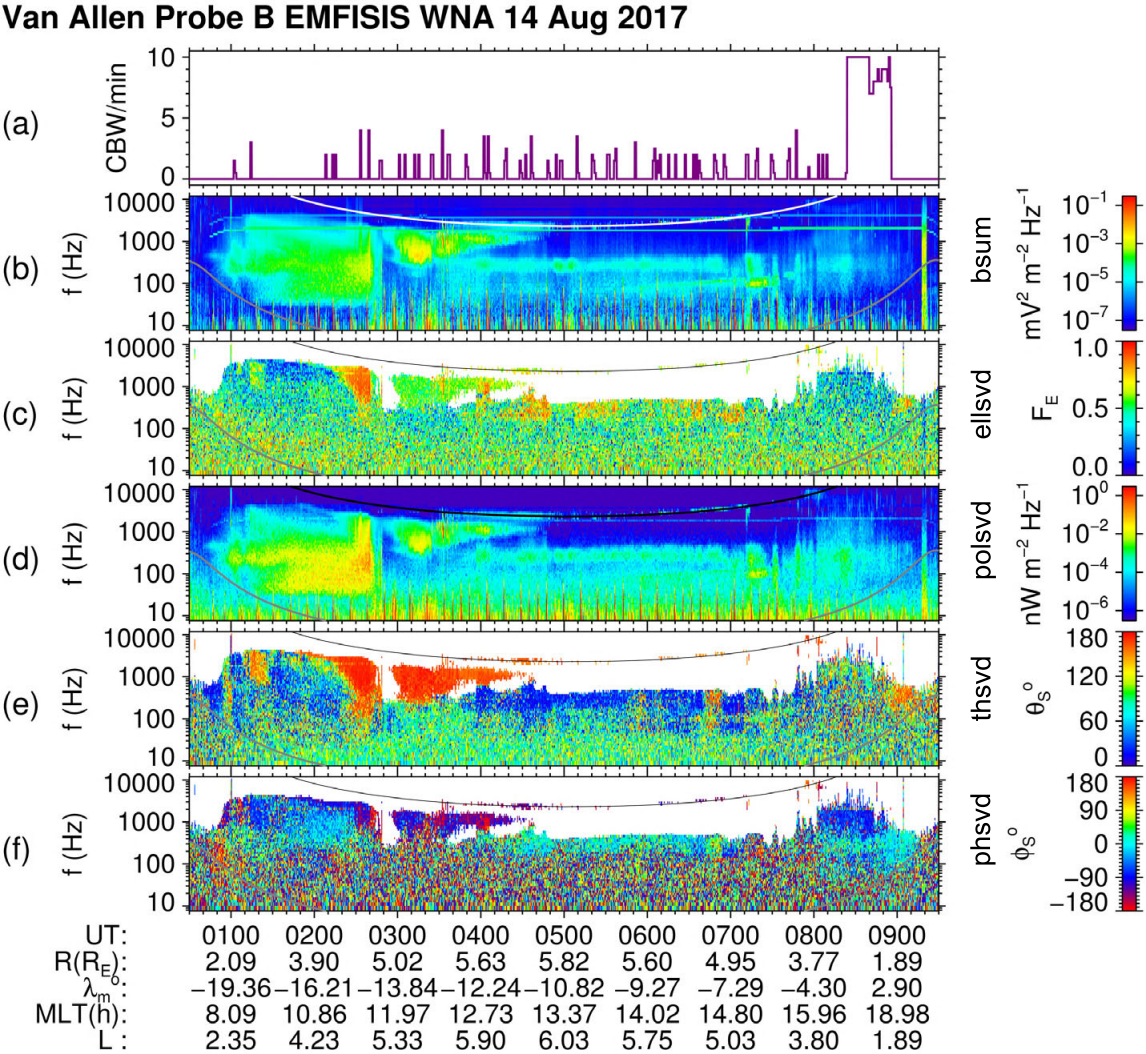
The same as in Fig. 11 but for another set of quantities from Table 3 in panels (**b**–**f**). Panel (**a**) shows the number of simultaneously recorded 6-s waveform captures per minute. An uninterrupted sequence of waveform captures have been obtained around 8:30 UT, see Miyoshi et al. this issue

Figure 12 shows an example of the results based on the combined magnetic and electric field measurements, introduced by a reference to the cadence of the simultaneously recorded 6 s long waveform captures in Fig. 12a, which demonstrates the complementarity of both data sets. The sequence capturing the lightning whistlers around 8:30 UT is analyzed from the waveform measurements by Santolík et al. (2021). The EMFISIS WNA data shown in the subsequent panels are again summarized in Table 3 with references to Fig. 12. These results show, for example, that the dawn side plasmaspheric hiss and exohiss/chorus emissions come with a high electromagnetic planarity and with the Poynting vector antiparallel to $\vec{B_{0}}$, i.e. from the equatorial region, on both inner and outer edge of the plasmapause density gradient. Exohiss close to the apogee propagates from the opposite direction, while lightning whistlers on the duskside come from both hemispheres.

#### Noise Floor

**Fig. 13:**
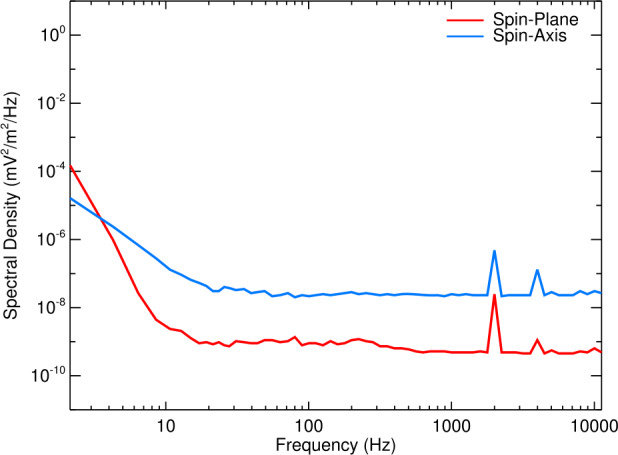
EMFISIS WFR electric field noise floor as a function of frequency. The red curve shows the noise floor for the spin-plane components and the blue curve shows the noise floor for the spin-axis component

In order to ensure that the EMFISIS WFR data has a scientifically useful signal, it is important to understand the signal-to-noise ratio (SNR). While examination of a given frequency-time plot gives a general sense of strong signals which are clearly evident, it is useful to be aware of the noise floor of the measurement for both electric and magnetic field components as a function of frequency. Figure 13 shows the electric field noise floor characterized during observation periods when there was little or no natural background signal. Note that there are two curves, one for the spin plane electric field probes (red curve) and another for the spin-axis component (blue curve). The two are different due to the much shorter length of the spin axis probes which inherently increases the background noise. These curves were generated from quiet periods throughout the mission and represent an average noise floor over the Van Allen Probes mission lifetime. The spikes around 2 and 4 kHz are interference lines (external to EMFISIS) which are quite narrow, but can move slightly in frequency. They are generally quite clear in line spectra or spectrograms.

**Fig. 14:**
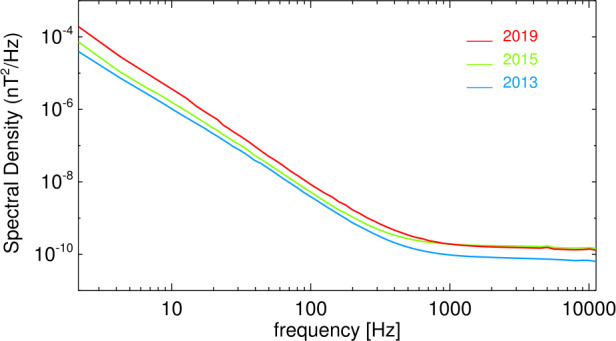
EMFISIS WFR MSC magnetic field noise floor as a function of frequency. The red curve shows the noise floor for all three components of the wave magnetic field. The colors indicated different periods of time and show that over the mission lifetime, the noise floor increased somewhat

Similar curves for the WFR MSC magnetic components are shown in Fig. 14. The different colors denote early in the mission (blue), mid-mission (green) and near the end of the mission (red) and show that over time, the noise floor did rise as the electronics aged. Note that all three axes of the MSC are identical, so this noise floor figure applies to any component. The increase of the noise floor at lower frequency arises because the MSC measures the change in $\vec{B}$ with respect to time ($d \vec{B}/dt$) and at lower frequencies, this derivative has a smaller magnitude.

As a general rule of thumb, signals which are at least an order of magnitude greater than the noise floors are distinct and scientifically useful. Smaller magnitude signals can be used, but care must be taken to understand the effect of the noise floor on those signals.

#### Noise Floor and Planarity

As discussed in Sects. 4.5.5 and 4.5.6, the planarity provides a useful metric for evaluating the results of the SVD process which gives the various WNA parameters. The noise floor, coupled with the frequency binning of the survey spectral matrix data, has an important effect on determining the level of the planarity required to have valid wave properties. This is because as the noise floor increases at lower wave frequencies, the ability to distinguish whether or not a given frequency bin yields a result which is consistent with a single plane wave description degrades significantly.

**Fig. 15:**
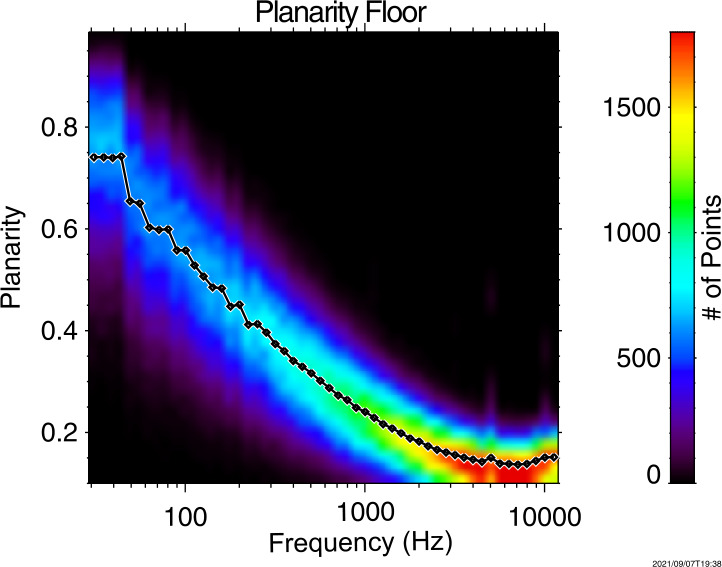
The level of planarity for EMFISIS spectral matrix survey data derived from instrumental background noise using the same SVD wave normal analysis that produces the EMFISIS L4 WNA data products. The line overplotted gives the median value of the noise planarity as a function of frequency

Figure 15 shows the planarity calculated from EMFISIS survey spectral matrix data for five very quiet intervals when instrument noise was essentially the only signal present. The median value of the planarity as a function of frequency is shown overplotted to help guide the eye. This figure shows the level at which the random background noise signal becomes indistinguishable from a plane wave as a function of frequency. As one can see, at frequencies above approximately 1 kHz, the noise planarity is around 0.2 and drops to a value of about 0.1 at the highest frequencies. Below 1 kHz, however, this curve rises to value of planarity near 0.6 at 100 Hz and up to almost 0.8 in the lower frequency bins. What this tells us is that a value of planarity of 0.6 at 100 Hz is consistent with random noise and is not an indicator of a reliable result wave parameter results.

This planarity noise floor rises at lower frequencies due to the combination of increasing instrument noise floor and, importantly, the decreasing number of FFT values which are averaged to produce the spectral matrix for that frequency as discussed in Sect. 4.5.1. With a large number of FFT values averaged together, the off-diagonal elements of the spectral matrix for a pure noise signal become more uncorrelated and the SVD determination of the eigenvalues which are used to compute planarity become much more distinct.

The key point for the reader is to be careful to use a sufficiently large value of planarity as indicated by Fig. 15 to ensure that the wave normal parameters are significant, that is, not consistent with noise, so that a valid scientific quantity results.

## The Waves Instrument: High Frequency Receiver (HFR) and Density

An important data set derived from the EMFISIS Waves data is that of the electron number densities. Waves normally measures the upper hybrid resonance frequency $f_{uh} = (f_{pe}^{2} + f_{ce}^{2})^{1/2}$ where $f_{pe}$ is the electron plasma frequency $f_{pe} = 8980 n_{e}^{1/2}$ and $f_{ce}$ is the electron cyclotron frequency $f_{ce}= 28|B|$ and where all frequencies are in Hz, the magnetic field is in nT, and densities are in $\text{cm}^{-3}$. In some cases, $f_{uh}$ is not apparent in the spectra, in which case the low-frequency cutoff of the continuum radiation can sometimes be used as a measure of $f_{pe}$. A detailed description of the techniques used in the interpretation of the Waves spectrum and the uncertainties in the resulting number density is given in Kurth et al. (2015).

**Fig. 16:**
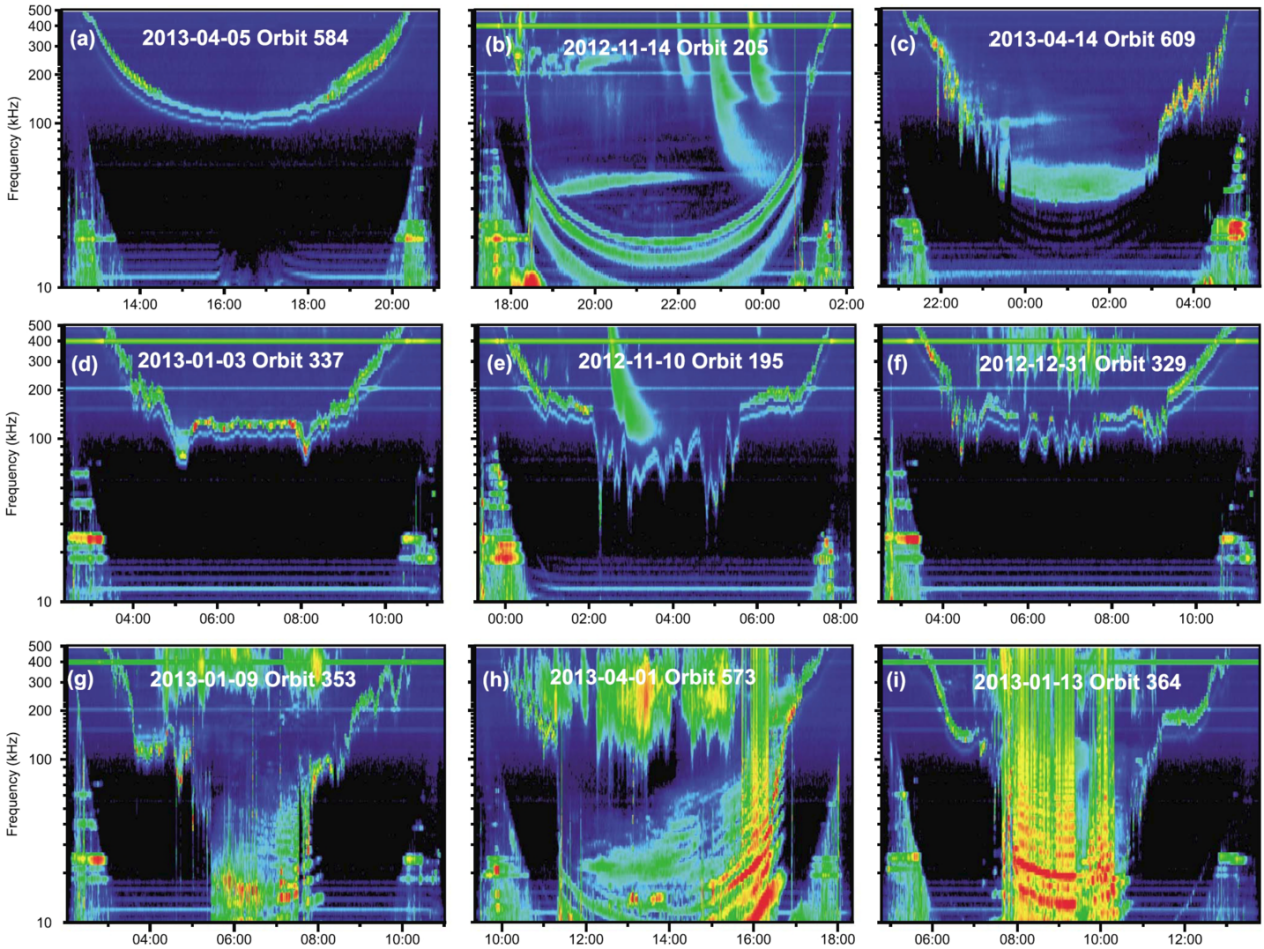
[From Kurth et al. 2015]: Examples of Van Allen Probe A Waves High Frequency Receiver spectrograms illustrating some of the varieties of forms the spectra take on depending on the level of activity of the magnetosphere. The inference of electron densities from the frequency of the upper hybrid resonance band relies on the proper identification of $f_{uh}$. See text for more discussion

Figure 16 is from Kurth et al. (2015) and is designed to show some of the variations observed in the Waves High Frequency Receiver (HFR) data that bear on the identification of $f_{uh}$ or $f_{pe}$. Roughly speaking, the complexity of the wave spectrum, hence, the difficulty of identifying the relevant feature for use in determining the electron density increases from Fig. 16a to 16i. In Fig. 16a, the spacecraft never exits the plasmasphere and the identification of $f_{uh}$ is relatively straightforward as the upper of the two bands. Kurth et al. (2015) used the cutoff of a type III solar radio burst to confirm that it is the upper of the two bands in Fig. 16a that is relevant to the frequencies near $f_{uh}$ and $f_{pe}$. While the lower band seems to show evidence of density variations, its nature is not well understood. Figure 16b is a somewhat similar situation showing a very simple spectrum, although in this case, the spacecraft exited the plasmasphere at about 18:30 on 14 November 2012 and reentered near 01:00 of the following day. There are electron cyclotron harmonics near $3f_{ce}/2$, $5f_{ce}/2$ and $7f_{ce} /2$ and we take the upper of these as the band closest to $f_{uh}$. In panel c, the plasmasphere is also eroded and there is no clear enhancement in the spectrum in the outer portion of this orbit that can be identified as $f_{uh}$. However, in this case, there is a clear lower cutoff of the trapped continuum radiation that gives an upper limit to $f_{pe}$. This is an upper limit to $f_{pe}$ because it could be that the continuum radiation is cutoff at a remote location and the density at the spacecraft is actually somewhat lower.

Panels d–f in Fig. 16 show rapid variations in the frequency of the upper hybrid band, hence the electron density. For these orbits, the interpretation of the spectrum is reasonably straightforward, but it is necessary to track the rapid excursions in frequency. Finally, panels g–i show deep erosions of the plasmasphere and very low densities beyond the plasmapause. These are indicative of an active magnetosphere and present the greatest challenge in the interpretation of the spectrum for the purposes of inferring the plasma density. As can be seen, there are multiple harmonics in the spectrum. These consist of multiple electron cyclotron harmonics between $f_{ce}$ and $f_{uh}$, a brighter band (usually) near $f_{uh}$, and even more harmonically-spaced bands at yet higher frequencies. Based on earlier work (Hubbard et al. 1979; Kurth et al. 1979) we take the brightest band above the first electron cyclotron harmonic as the one closest to $f_{uh}$. The higher frequency bands may be Fq resonances or may be due to instrumental distortion at high signal levels.

As described in Kurth et al. (2015) an algorithm called Automated Upper-hybrid Resonance detection Algorithm (AURA) was developed to semi-automate the identification of the upper hybrid band in the Waves spectral data. We found that AURA worked quite well at tracking $f_{uh}$ in situations like those depicted in Fig. 16 panels a–b and d–f. For such orbits, it usually sufficed to do a quick manual inspection of the algorithm results before using them to determine electron densities. The active orbits, like those in panels g–i almost always required manual operation of the software to achieve acceptable identification of the upper hybrid band. And, even with manual intervention, the proper identification of $f_{uh}$ was possibly either suspect or not possible. Such questionable time intervals are often not represented in the resulting density data set because of the inability to reliably identify $f_{uh}$. The basic uncertainty in the Waves electron density data set is based on the spectral resolution of the High Frequency Receiver which is $\Delta f/f \sim 5\%$. Since $n_{e}$ is proportional to $f_{uh}^{2}$, the fundamental uncertainty in $n_{e}$ is $\sim 10$%. However, a more important source of error for the data set has to do with the proper identification of the upper hybrid band. As shown by Kurth (1982), as $f_{pe}$ varies from one electron cyclotron harmonic to another, the intensified band near $f_{uh}$ steps from one harmonic to another, as well. If $f_{uh} \gg f_{ce}$, then this difference is relatively small. However, if $f_{uh}$ is in one of the lower cyclotron harmonic bands, $f_{uh}$ is known no better that $\delta f_{ce}/f_{uh}$ which can be a significant fraction for low harmonic bands. Worse, if the appropriate harmonic band is misidentified, the error is, again, separated by at least one if not more factors of $f_{ce}$. Hence, during active times when the magnetosphere beyond the plasmapause has a low density, the spectrum is difficult to interpret and relative errors can become large. The resulting density values can certainly indicate that these regions have very low densities, but an accurate measure can be problematic.

There have been additional efforts to use the EMFISIS Waves spectrum to determine the electron density. One of them (Zhelavskaya et al. 2016) uses a neural network to identify the upper hybrid frequency in the Waves data. The neural network was trained using previously determined values of $f_{uh}$ based on Kurth et al. (2015). Another effort, by Hartley et al. (2017, 2018a) uses cold plasma dispersion theory and the observed ratio of electric and magnetic field wave power of plasmaspheric hiss to infer the plasma density. This technique yields densities comparable to those derived from $f_{uh}$ and, notably, provides densities deep in the plasmasphere where the upper frequency limit of the Waves High Frequency Receiver does not permit observations of $f_{uh}$. Recently, Jahn et al. (2020) compared electron densities obtained from $f_{uh}$ and from the spacecraft potential used as a proxy for the plasma density. Interestingly, this represents a full-circle return to the earlier work by Kurth et al. (2015) in that spacecraft potential measurements by the Van Allen Probes Electric Fields and Waves (EFW) investigation (Wygant et al. 2013, S. Thaller, personal communication, 2013) were used intensively to validate the early $f_{uh}$ results. As pointed out by Jahn et al. (2020), the empirical relation between spacecraft potential and plasma density can vary with environmental effects and changes in the probe, such as the work function of the probe coating material. This results in a bootstrap relation between the two techniques where the $f_{uh}$ technique provides a calibration for the spacecraft potential proxy, but the potential measurement is often available when $f_{uh}$ is not available or uncertain.

## Conclusion

The EMFISIS instrumentation has produced one of the most complete sets of DC magnetic field and wave data in the inner magnetosphere ever made. The DC magnetic field data have provided excellent determination of low frequency waves and accurate determination of pitch angle for the particle instruments.

Of particular note is the full 3D vector measurements of the wave electric and magnetic field over the key range of frequencies covering chorus waves and lower frequency phenomena which has supported a wide range of scientific discovery on NASA’s Van Allen Probes. This has enabled the determination of key wave properties such as polarization, ellipticity, Poynting flux, and wave normal direction on a regular basis. When coupled with the large volume of burst mode waveform data, the calculation of these quantities with unprecedented resolution has been achieved.

The measurement of higher frequency wave electric fields has produced observation of the upper hybrid line excited by the thermal background to yield a regular determination of plasma density. Indeed, the EMFISIS investigation has produced the most complete set of plasma density measurements ever made in the inner magnetosphere. This key plasma parameter is fundamental to accurate theoretic calculations and has proved invaluable for many studies.

The Van Allen Probes mission has been extremely successful and the EMFISIS team has been proud to participate.

## Appendix A: Mathematics of $\vec{E}\cdot \vec{B}$ for Spectral Components

In working out the corrections for sheath impedance, it became clear that synthesis of the axial (W) component of the electric field was important as a check for the corrections. However, this requires a methodology for deriving the axial component in the frequency domain rather than in the directly sampled signal. What follows is that mathematics of how this was achieved.

We start with Faraday’s law:

28 $$ \boldsymbol{\nabla} \times \vec{E} = -\partial \vec{B}/ \partial t $$

Making the plane wave assumption that:

$$\begin{aligned} \vec{E}, \vec{B} \propto \exp{j(\vec{k} \cdot \vec{r} - \omega t)} \end{aligned}$$

we arrive at the following form for Faraday’s law:

29 $$ \vec{k} \times \vec{E} = \omega \vec{B}, $$

where $\vec{k}$ denotes the wave vector, $\omega $ denotes the angular frequency, and $\vec{E}$ and $\vec{B}$ denote the electrical and magnetic fields respectively.

Since $\vec{k}$ is assumed to be real-valued, we may write $\vec{k} = \vec{k_{r}}$, where $\vec{k_{r}} \in \Re ^{3}$, where $\Re ^{3}$ denotes the three-dimensional Euclidean vector space over the field of real numbers. Now breaking up the other vectors $\vec{E}$ and $\vec{B}$ in Eq. (29) into real and imaginary parts, we get:

30 $$ \vec{E} = \vec{E}_{r} + j \vec{E}_{i},\quad \mbox{where $\vec{E_{r}} \in \Re ^{3}$, $\vec{E_{i}} \in \Re ^{3}$, and} $$

31 $$ \vec{B} = \vec{B}_{r} + j \vec{B}_{i},\quad \mbox{where $\vec{B_{r}} \in \Re ^{3}$, $\vec{B_{i}} \in \Re ^{3}$.} $$

Taking the real and imaginary parts on all vectors in Eq. (29) we get:

32 $$ \vec{k_{r}} \times \left ( \vec{E}_{r} + j \vec{E}_{i}\right ) = \omega \left ( \vec{B}_{r} + j \vec{B}_{i} \right ), $$

33 $$ \left ( \vec{k_{r}} \times \vec{E}_{r} \right ) + j \left ( \vec{k_{r}} \times \vec{E}_{i}\right ) = \omega \vec{B}_{r} + j \omega \vec{B}_{i} . $$

Equating the real and imaginary parts on both sides of Eq. (33) we get:

34 $$ \vec{k}_{r} \times \vec{E}_{r} = \omega \vec{B}_{r}, \mbox{ and } $$

35 $$ \vec{k}_{r} \times \vec{E}_{i} = \omega \vec{B}_{i}. $$

Therefore, the fact that $\vec{k} = \vec{k}_{r} \in \Re ^{3}$ can be exploited to break up Eq. (29) into the independent Eqs. (34) and (35).

### Remark 1

Equations (34) and (35) express the relationship between the purely real or imaginary parts of $\vec{E}$ and $\vec{B}$ respectively, which individually hold but do not offer any interpretation of the cross-terms involving the real and imaginary parts, e.g. between $\vec{E}_{r}$ and $\vec{B}_{i}$, etc. However, a full analysis of (29) necessitates the investigation of such cross-terms. Accordingly, we present the detailed calculation below to ensure consistency between Eqs. (34) and (35) as the purely real and purely imaginary interpretation of Eq. (29), and also to derive corresponding relationships between the cross-terms consistent with Eq. (29).

Taking the dot product with respect to $\vec{E}_{r}$ on both sides of Eq. (34) yields:

36 $$ \vec{E}_{r} \cdot \left ( \vec{k}_{r} \times \vec{E}_{r} \right ) = \omega \vec{E}_{r} \cdot \vec{B}_{r} = 0. $$

Similarly, we also have:

37 $$ \vec{E}_{i} \cdot \left ( \vec{k}_{r} \times \vec{E}_{i} \right ) = \omega \vec{E}_{i} \cdot \vec{B}_{i} = 0. $$

We will check this result in Eqs. (36) and (37) for consistency with Eq. (38) below that takes the dot product of Eq. (29) with respect to $\vec{E}$.

38 $$ \vec{E} \cdot \left ( \vec{k} \times \vec{E} \right ) = \omega \vec{E} \cdot \vec{B} = 0. $$

Breaking up Eq. (38) into real and imaginary parts on both sides, we get:

39 $$ \left ( \vec{E}_{r} + j \vec{E}_{i} \right ) \cdot \left ( \vec{k}_{r} \times \left ( \vec{E}_{r} + j \vec{E}_{i} \right ) \right ) = \omega \left ( \vec{E}_{r} + j \vec{E}_{i} \right ) \cdot \left ( \vec{B}_{r} + j \vec{B}_{i} \right ) = 0. $$

Separating the real and imaginary parts on both sides of Eq. (39) leads to:

40 $$ \vec{E}_{r} \cdot \left ( \vec{k}_{r} \times \vec{E}_{r} \right ) - \vec{E}_{i} \cdot \left ( \vec{k}_{r} \times \vec{E}_{i} \right ) = \omega \left ( \vec{E}_{r} \cdot \vec{B}_{r} - \vec{E}_{i} \cdot \vec{B}_{i} \right ) = 0, \mbox{ and } $$

41 $$ \vec{E}_{i} \cdot \left ( \vec{k}_{r} \times \vec{E}_{r} \right ) + \vec{E}_{r} \cdot \left ( \vec{k}_{r} \times \vec{E}_{i} \right ) = \omega \left ( \vec{E}_{i} \cdot \vec{B}_{r} + \vec{E}_{r} \cdot \vec{B}_{i} \right ) = 0 $$

We note that Eq. (40) is consistent with Eqs. (36) and (37), which were derived from Eqs. (34) and (35) respectively. Equation (41) represents the cross-terms between real and imaginary parts of $\vec{E}$ and $\vec{B}$ and as such are not relevant to Eqs. (34) and (35), which represent the expressions between the purely real (or imaginary) components only for $\vec{E}$ and $\vec{B}$.

We can derive the following relationships between the cross-terms involving the real and imaginary parts of $\vec{E}$ and $\vec{B}$ based on Eq. (41).

42 $$ \vec{E}_{i} \cdot \left ( \vec{k}_{r} \times \vec{E}_{r} \right ) = - \vec{E}_{r} \cdot \left ( \vec{k}_{r} \times \vec{E}_{i} \right ), \mbox{ and } $$

43 $$ \vec{E}_{i} \cdot \vec{B}_{r} = - \vec{E}_{r} \cdot \vec{B}_{i} . $$

## Appendix B: EMFISIS Key Specifications

### B.1 Magnetometer

Cadence: 64 vectors/s

**Table:**

Range ID	Field range	Resolution	Accuracy (sensor only)	Accuracy (deployed on spacecraft)
3	±65536 nT	2 nT	0.1 nT	5 nT
1	±4096 nT	0.16 nT	0.1 nT	5 nT
0	±256 nT	0.001 nT	0.1 nT	5 nT

### B.2 Waves

#### B.2.1 WFR Receiver Performance

MSC Noise Floor best estimate from lab calibrations:

(Noise levels below 100 Hz are difficult to ascertain due to 60 Hz interference)

**Table:**

Frequency	Noise level
100 Hz	$< 10^{-8}~\text{nT}^{2}/\text{Hz}$
1 kHz	$< 10^{-10}~\text{nT}^{2}/\text{Hz}$
10 kHz	$< 5\times 10^{-10}~\text{nT}^{2}/\text{Hz}$

Dynamic Range: 96 dB (19 dB switchable attenuator)

Electric field Noise Floor (100 meter baseline) best estimate from lab calibrations:

**Table:**

Frequency	Noise level
2.5 Hz	$< 10^{-14}~\text{(V/m)}^{2}/\text{Hz}$
10 Hz	$< 10^{-15}~\text{(V/m)}^{2}/\text{Hz}$
100 Hz	$< 5\times 10^{-15}~\text{(V/m)}^{2}/\text{Hz}$
1 kHz	$< 5\times 10^{-16}~\text{(V/m)}^{2}/\text{Hz}$
10 kHz	$< 5\times 10^{-16}~\text{(V/m)}^{2}/\text{Hz}$

#### B.2.2 HFR Receiver Performance

Noise Floor (100 meter baseline) best estimate from lab calibrations:

**Table:**

Frequency	Noise level
10 kHz	$<10^{-17}~\text{(V/m)}^{2}/\text{Hz}$
100 kHz	$<10^{-17}~\text{(V/m)}^{2}/\text{Hz}$
500 kHz	$< 2 \times 10^{-17}~\text{(V/m)}^{2}/\text{Hz}$

Dynamic Range: 84 dB (19 dB switchable attenuator)

### B.3 Standard Survey Products

Note that in what follows the names used are those which correspond to the data products rather than the names of the modes described above. There is a close correspondence, but for data users, the names below match those of files containing the data that is described.

#### B.3.3 HFR-Spectra

$E_{U}$ or $E_{V}$ or $E_{W}$:

Cadence: 6 s 4096 samples at 1 MSamples per second (14 bit digitization)

Bin averaged into 82 logarithmically spaced bins between 10 kHz to 500 kHz

#### B.3.4 WFR-Spectral-Matrix

Three Axis Electric Field ($E_{U}, E_{V}, E_{W}$) and Three Axis Magnetic field ($B_{U}, B_{V}, B_{W}$) cross spectral matrix, 6 diagonal components and 15 off-diagonal components:

$$\begin{aligned} \textstyle\begin{array}{l@{\ \ }|@{\ \ }l@{\quad}l@{\quad}l@{\quad}l@{\quad}l@{\quad}l} & E_{U} & E_{V} & E_{W} & B_{U} & B_{V} & \mathbf {B}_{W}\\ \hline E_{U} & E_{U}E_{U} & E_{U}E_{V} & E_{U}E_{W} & \text{--} & \text{--} & \text{--} \\ E_{V} & \text{--} & E_{V}E_{V} & E_{V}E_{W} & \text{--} & \text{--} & \text{--} \\ E_{W} & \text{--} & \text{--} & E_{W}E_{W} & \text{--} & \text{--} & \text{--} \\ B_{U} & B_{U}E_{U} & B_{U}E_{V} & B_{U}E_{W} & B_{U}B_{U} & B_{U}B_{V} & B_{U}B_{W} \\ B_{V} & B_{V}E_{U} & B_{V}E_{V} & B_{V}E_{W} & \text{--} & B_{V}B_{V} & B_{V}B_{W} \\ B_{W} & B_{W}E_{U} & B_{W}E_{V} & B_{W}E_{W} & \text{--} & \text{--} & B_{W}B_{W} \\ \end{array}\displaystyle \end{aligned}$$

Units:

$$\begin{aligned} \textstyle\begin{array}{l@{\ \ }|@{\ \ }l@{\quad}l@{\quad}l@{\quad}l@{\quad}l@{\quad}l} & E_{U} & E_{V} & E_{W} & B_{U} & B_{V} & \mathbf {B}_{W}\\ \hline E_{U}& \text{(V/m)}^{2}/\text{Hz} & \text{(V/m)}^{2}/\text{Hz} & \text{(V/m)}^{2}/\text{Hz} & \text{--} & \text{--} & \text{--} \\ E_{V} & \text{--} & \text{(V/m)}^{2}/\text{Hz} & \text{(V/m)}^{2}/\text{Hz} & \text{--} & \text{--} & \text{--} \\ E_{W} & \text{--} & \text{--} & \text{(V/m)}^{2}/\text{Hz} & \text{--} & \text{--} & \text{--} \\ B_{U} & \text{(nT-V/m)/Hz} & \text{(nT-V/m)/Hz} & \text{(nT-V/m)/Hz} & \text{nT}^{2}/\text{Hz} & \text{nT}^{2}/\text{Hz} & \text{nT}^{2}/\text{Hz} \\ B_{V} & \text{(nT-V/m)/Hz} & \text{(nT-V/m)/Hz} & \text{(nT-V/m)/Hz} & \text{--} & \text{nT}^{2}/\text{Hz} & \text{nT}^{2}/\text{Hz} \\ B_{W} & \text{(nT-V/m)/Hz} & \text{(nT-V/m)/Hz} & \text{(nT-V/m)/Hz} & \text{--} & \text{--} & \text{nT}^{2}/\text{Hz}\\ \end{array}\displaystyle \end{aligned}$$

Cadence: 6 s ($E_{U}, E_{V}, E_{W}, B_{U}, B_{V}, B_{W}$)

16384 samples at 35 kSamples per second (16 bit digitization)

Bin averaged into 65 logarithmically spaced bins between 2 Hz to 12 kHz

#### B.3.5 WFR-Waveform

6 channels ($E_{U}, E_{V}, E_{W}, B_{U}, B_{V}, B_{V}$)

Cadence: ≈once per 15 minutes

16384 samples at 35 kSamples per second (16 bit digitization)

### B.4 Waves Burst Mode Products (Commandable and Limited by Memory Allocations)

#### B.4.6 HFR-Waveform

Cadence: commandable (not a standard product)

4096 samples at 1 MSamples per second (14 bit digitization)

#### B.4.7 HFR-Spectra-Burst

$E_{U}$ or $E_{V}$ or $E_{W}$

Cadence: 0.5 s

4096 samples at 1.325 MSamples per second (14 bit digitization)

Bin averaged into 82 logarithmically spaced bins between 10 kHz to 500 kHz

#### B.4.8 WFR-Spectral-Matrix-Burst

Similar to the standard survey product described above.

Cadence: 1 s

16384 samples at 35 kSamples per second (16 bit digitization)

Bin averaged into 65 logarithmically spaced bins between 2.14 Hz to 11.2 kHz

#### B.4.9 WFR-Spectral-Matrix-30 msec

6 channels

Cadence: 0.5 s

16 sets of 1024 samples at 35 kSamples per second (16 bit digitization)

#### B.4.10 WFR-Spectra-30 msec-Mode

6 channels ($E_{U}, E_{V}, E_{W}, B_{U}, B_{V}, B_{V}$)

Cadence: 30 msec

1024 samples at 35 kSamples per second (16 bit digitization)

Bin averaged into 70 linearly spaced bins (170 Hz band width) between 102.5 Hz to 11.894 kHz

#### B.4.11 Wave Normal Analysis Mode (WNA Mode)

Onboard processing of wave normal parameters based on 30 msec mode spectral matrix data.

As a function of frequency:

- $B^{2}$ – Squared magnetic field magnitude, $\text{nT}^{2}/\text{Hz}$
- $E^{2}$ – Squared electric field magnitude, $\text{(V/m)}^{2}/\text{Hz}$
- $S_{x}$ – Poynting Vector, $\text{W}/\text{m}^{2}$
- $S_{y}$ – Poynting Vector, $\text{W}/\text{m}^{2}$
- $S_{z}$ – Poynting Vector, $\text{W}/\text{m}^{2}$
- Magnetic eigenvectors and eigenvalues – arbitrary units with component values for vectors.

#### B.4.12 WFR-Waveform-Burst

6 channels ($E_{U}, E_{V}, E_{W}, B_{U}, B_{V}, B_{V}$)

16384 samples at 35 kSamples per second (16 bit digitization) for 0.468 s

#### B.4.13 WFR-Waveform-Continuous-Burst

6 channels ($E_{U}, E_{V}, E_{W}, B_{U}, B_{V}, B_{V}$)

208896 samples at 35 kSamples per second (16 bit digitization) for 5.968 s
